# Interventions for the prevention of weight gain during festive and holiday periods in children and adults: A systematic review

**DOI:** 10.1111/obr.13836

**Published:** 2024-09-14

**Authors:** Diego E. Guerrero‐Magaña, Lucía G. Urquijo‐Ruiz, Alma L. Ruelas‐Yanes, Teresita de J. Martínez‐Contreras, Rolando G. Díaz‐Zavala, Maria del Carmen Candia‐Plata, Julián Esparza‐Romero, Michelle M. Haby

**Affiliations:** ^1^ Posgrado en Ciencias de la Salud, Facultad Interdisciplinaria de Ciencias Biológicas y de la Salud Universidad de Sonora Hermosillo Sonora Mexico; ^2^ Departamento de Ciencias Químico‐Biológicas, Facultad Interdisciplinaria de Ciencias Biológicas y de la Salud Universidad de Sonora Hermosillo Sonora Mexico; ^3^ Departamento de Medicina y Ciencias de la Salud, Facultad Interdisciplinaria de Ciencias Biológicas y de la Salud Universidad de Sonora Hermosillo Sonora Mexico; ^4^ Unidad de Investigación en Obesidad y Diabetes, Coordinación de Nutrición, Centro de Investigación en Alimentación y Desarrollo (CIAD), Carretera Gustavo Enrique Astiazarán Rosas Hermosillo Sonora Mexico; ^5^ Melbourne School of Population and Global Health The University of Melbourne Parkville Victoria Australia

**Keywords:** festive periods, holiday season, obesity, prevention, summer, systematic review

## Abstract

Some periods during the year, such as festive and summer holiday periods, have been associated with weight gain. We aimed to assess the effect of interventions for the prevention of body weight gain during festive and holiday periods in children and adults. A systematic search was conducted in six databases and supplementary sources until January 4, 2023. We included randomized controlled trials (RCTs), cluster‐RCTs, and non‐RCTs. Our primary outcome measure was the change in body weight in adults or the change in BMI z‐score or BMI percentile in children and adolescents. From 4216 records, 12 primary studies (from 22 reports) met the inclusion criteria—10 from the United States, one from the United Kingdom, and one from Chile. Two studies had a low risk of bias, two moderate, seven high, and one critical risk of bias. The meta‐analysis in children included four of seven studies during the summer holidays (six interventions) and showed a mean difference in BMI z‐score favoring the intervention group (−0.06 [95% CI −0.10, −0.01], *p* = 0.01, *I* ^2^ = 0%, very low certainty evidence). The meta‐analysis in adults included five studies during festive periods with a mean difference in weight favoring the intervention group (−0.99 kg [95% CI −2.15, 0.18], *p* = 0.10, *I* ^2^ = 89%, very low certainty evidence). This review has highlighted potential interventions to prevent the increase in body weight during holiday periods. More work is needed to improve the quality of the evidence and to extend it to countries outside of the United States and United Kingdom and to the adolescent population.

## 1 INTRODUCTION

Obesity is one of the main health problems in the world. There is a large body of evidence that shows a causal relationship between excess body weight and chronic diseases such as type 2 diabetes, cardiovascular disease, and certain types of cancer, among others.^1^ In 2016, it was reported that about 124 million boys and girls; and approximately 671 million men and women suffered from obesity globally.^2^ Furthermore, the contribution of high body mass index to the global burden of disease is increasing.^1^

One way to treat obesity is using an intensive lifestyle intervention that aims to achieve a weight reduction of 10% in 12 months.^3^ However, in the long‐term, some of the positive effect on weight is lost.^4^ This is due to the difficulty of achieving substantial and permanent changes in diet and physical activity in an obesogenic environment.^5^, ^6^ In addition, there are neuroendocrine changes accompanying weight loss that favor weight regain.^7^, ^8^, ^9^ Pharmacological treatment of obesity has improved dramatically in recent years;^10^ however, patients often regain most of the weight lost upon discontinuation of the drug.^11^ For this reason, interventions to prevent weight gain are likely to be a better strategy than treating obesity once it is established.

There is evidence that some periods during the year, such as festive and summer holiday periods, are associated with weight gain.^12^, ^13^, ^14^, ^15^ For example, it has been suggested that there may be an accelerated gain in weight in children and adolescents during the summer school holidays and that this may especially affect high‐risk groups, such as certain racial populations (black, Hispanic) and those with overweight or obesity.^14^ For children, for example, a 5‐year follow‐up of 7599 ethnically diverse participants from 41 elementary schools in the United States showed a 0 to 0.06 BMI‐z‐score decrease during school months, whereas increases of BMI‐z‐score ranging from 0.04 to 0.09 were observed during the summer.^16^ The authors noted that the mechanisms for the larger weight gain during the summer period than during school time need further research and were not measured as part of their study. However, they hypothesized that they could be due to decreases in physical activity, eating in front of the television, and disruptions in household routines, including sleep patterns, meal times and snacking habits.^16^

For adults, a recent narrative review by our research group found that significant increases in body weight have been reported in various observational studies, ranging from 0.4 kg to 0.9 kg during the 6–8 week period of the December holidays (between Thanksgiving and New Year, which is winter time in the northern hemisphere).^12^ This weight gain represents a significant proportion of the total annual weight gain. For example, a study with a 1 year follow‐up of 195 adults in the United States showed that the weight gained in the 6–8 week December holiday period represented more than 50% of the total weight gained throughout the year and that this weight was not subsequently lost.^13^ Further, although the increase in weight during the holiday period appears to be small numerically, in 10 years it could represent an overall increase of 5 to 10 kg^17^ across the adult population. Possible mechanisms discussed by the authors of these studies include an increase in consumption of energy dense foods, relaxed eating times and habits, and a decrease in physical activity.^12^, ^13^, ^17^

Interventions to prevent weight gain during festive and summer holiday periods are needed. A systematic scoping review by Zorbas and colleagues, published in 2020, included 39 studies, of which six were studies of interventions for the prevention of body weight gain during the festive periods, with most of them focusing on the December holiday season.^15^ This scoping review found that 70% of 23 observational studies that measured changes in body weight showed significant increases during festive periods (reported mean of 0.7 kg). The six intervention studies (five RCTs and one pre‐post study), all with adults, showed that increases of body weight can be ameliorated by interventions such as self‐weighing and intermittent fasting. They noted that intervention groups consistently maintained or lost weight, while placebo or comparator groups (in the case of the five RCTs) increased weight or did not significantly change weight.^15^ However, given that it was a scoping review, it was not designed to evaluate the effectiveness of the interventions, and it did not include an assessment of the risk of bias of the included studies. Further, the date of last search for studies was March 2019. The only other systematic review of the effect of the holiday season was conducted in 2014 and is limited to children and the summer holiday period.^14^ The main objective of the review was to examine variations in weight gain during the summer holidays in comparison to the school year, focusing on racial/ethnic disparities. This systematic review included seven studies, and highlighted, as potential solutions for mitigating summer weight gain, interventions such as access to recreational spaces, physical activity, and food service programs. Limitations of this systematic review include the searching of two databases only (PubMed and EMBASE), only including studies published in peer review journals in English, and the lack of assessment of the risk of bias of included studies. Further, there were no limits to the study designs included and a mix of observational and quasi‐experimental studies are described—none were RCTs or included a control group. Finally, the date of last search was over 10 years ago (August 2013).^14^

To overcome these limitations, we conducted a systematic review with the aim to assess the effectiveness of interventions for the prevention of body weight gain during the festive or holiday periods in children and adults. We included both holiday types and both age groups in the one review for several reasons: (1) the determinants of weight gain are likely to be similar in both age groups (i.e., a decrease in physical activity, an excess in food consumption, and dysregulation in sleep patterns); (2) we hoped to find studies conducted in the southern hemisphere where the December holiday period coincides with the summer holidays; and (3) both the review process, reporting and interpretation of the results would be more efficient and useful than if the review was split into two.

## 2 MATERIALS AND METHODS

The protocol for this systematic review was registered on the International prospective register of systematic reviews (PROSPERO: CRD42020218883).^18^ The methods used were based on the Cochrane handbook for systematic reviews of interventions, and the reporting follows the Preferred Reporting Items for Systematic Reviews and Meta‐Analysis (PRISMA) guidelines.^19^, ^20^

### 2.1 Eligibility criteria

We included both published and unpublished (gray literature) studies in any language, with no date of publication limitations.

#### 2.1.1 Population

Persons aged 6 years and over with an initial BMI greater than 18.5 kg/m^2^ (or equivalent in children and adolescents) were included in the review. To be included in the review, studies needed to have a primary focus on prevention of weight gain rather than weight loss. Studies that included only people with diagnosed diabetes, uncontrolled hypertension, pregnant, or breastfeeding women or that used medications with an effect on body weight were excluded, as were studies conducted in an in‐patient setting.

#### 2.1.2 Intervention

Studies aimed at preventing an increase in body weight during the festive or holiday periods with a minimum duration of intervention of 2 weeks were included. Interventions could include dietary advice, behavior change, implementation of exercise or physical activity, strategies to reduce sedentary behavior, self‐monitoring strategies (e.g., self‐weighing), supplements, intermittent fasting, or any other strategy that was focused on the prevention of weight gain. The interventions could be delivered in person, web‐based, or any combination of these. Studies that had a goal of weight loss, which is an aim of treatment of overweight or obesity, were excluded. Studies focused exclusively on sedentary lifestyle patterns that were not aimed at preventing weight gain were also excluded. If the studies contained multiple intervention arms, the arms that met the inclusion criteria were included in the review.

#### 2.1.3 Comparison

Minimal intervention (e.g., nutrition information presented in a leaflet or flyer), usual care, waiting list, no intervention, or placebo interventions that were not related to the prevention of body weight gain.

#### 2.1.4 Study designs

Randomized controlled trials (RCT), cluster‐randomized controlled trials (cluster‐RCT), and nonrandomized controlled trials (non‐RCT) were included.

### 2.2 Outcomes

Our primary outcome measure was the change in body weight in adults or the change in BMI z‐score or BMI percentile in children and adolescents. Secondary outcome measures were adverse effects (regardless of how they were reported), change in BMI (adults only), body fat, and waist circumference in children and adults.

We did not exclude trials in the case that one or more of our primary or secondary outcome measures were not reported in the publication. When none of our primary or secondary variables were reported, the authors were contacted to try to obtain the missing data. If no relevant outcome data were obtained, the trials were still included. This decision is in line with the recommendation made in section 3.2.4.1 of the Cochrane handbook.^21^ This is done to avoid bias arising from selective reporting of findings by the study authors. Due to the nature of the interventions, however, most studies did in fact measure weight, though it was not always reported in the publication or in a form that could be included in the meta‐analysis (see results).

### 2.3 Search strategy

The following databases were searched from the inception of the database until the present date: Medline (Ovid), EMBASE (Ovid), LILACS, PsycINFO (Ovid), SciELO, and the Cochrane Central Register of Controlled Trials (CENTRAL) (Ovid). Search terms included Medical Subject Headings (MeSH), where applicable, and text words in the title, abstract and keywords fields. The population, interventions, comparison, outcomes, and study (PICOS) type framework and other systematic reviews on the topic were used to identify relevant search terms. In practice, the search strategy included terms related to the concepts: holiday types, intervention types, intervention aim (prevention of weight gain), and study type. The search strategies for each database can be found in File S1.

Reference lists of included studies and systematic reviews already completed were searched for relevant articles. Clinical trial registries were also searched, including ClinicalTrials.gov (https://clinicaltrials.gov/) and the World Health Organization (WHO) International Clinical Trials Registry Platform (ICTRP) search portal (http://apps.who.int/trialsearch/). In addition to the gray literature identified from the above sources, a Google Search was performed using the same keywords. The main authors of the included studies and key researchers in the field were contacted to identify both published and unpublished studies that we may have missed.

### 2.4 Study selection

References were imported into Endnote and duplicates were removed before screening. The initial analysis of titles and abstracts was conducted by two authors independently (LGUR and DEGM). The full‐text articles of any potentially relevant papers identified by either reviewer were obtained for closer examination. The inclusion criteria were applied against each of these papers by two reviewers independently (DEGM and [LGUR, ALRY, or TJMC]). Differences were resolved by discussion and consensus. A third author was consulted if any doubts remained (MMH). If necessary, the authors of the trial were contacted for clarification and in case of missing information. Studies excluded at this stage were reported in a table, along with the reason for their exclusion.

### 2.5 Data extraction

Data were extracted by two authors (ALRY and DEGM) independently. All disagreements were resolved by discussion and consensus or, if required, by consultation with a third reviewer (MMH). Data were extracted into a Microsoft Excel spreadsheet and included study information (author/s, year and country of study, study design, festive or holiday period studied, setting, and study duration (follow‐up)); details of participants (sample size, age range, race/ethnicity, socioeconomic status); interventions (intensity and components of the intervention and comparison groups—described according to the “Template for intervention description and replication” [TIDieR] checklist^22^); primary and secondary outcomes measured; and key findings. Numerical data on effectiveness results and adverse effects were reported using standard data extraction templates in Microsoft Excel.

### 2.6 Dealing with duplicate and companion publications

For duplicate publications, companion papers, or multiple reports from a primary trial, information yield was maximized through the collection of all available data, and the most comprehensive dataset for the study was obtained from all known publications. The paper with the most complete information was designated the primary reference for the study. Duplicate publications, supporting documents, multiple reports from a primary trial, and trial documents from included trials (such as trial registration information) are listed as secondary references under the ID of the included study.

### 2.7 Assessment of risk of bias in included studies and quality of the evidence

Two reviewers (ALRY and DEGM) independently assessed the risk of bias for each of the included studies. Discrepancies were resolved through discussion or consultation with a third author (MMH). The revised Cochrane Excel tool for assessing risk of bias (RoB 2.0) for individual randomized trials^23^ was used and the risk of bias criteria were judged as “low risk,” “high risk,” or “some concerns.” The individual items of bias were evaluated as described in the Cochrane Handbook for Systematic Reviews of Interventions^21^ and in the additional materials provided on the tool website (https://www.riskofbias.info/). Our effect of interest was the effect of assignment to intervention or comparison group. The primary outcomes (change in body weight in adults or the change in BMI z‐score or BMI percentile in children and adolescents) were assessed for risk of bias in included studies. For cluster‐RCTs, the revised RoB 2.0 with additional considerations for cluster‐RCTs (RoB 2 CRT) Excel tool was used.^24^ For non‐RCTs the ROBINS‐I tool was used,^25^ with age and sex pre‐identified as important confounders.

The Grading of Recommendations Assessment, Development, and Evaluation (GRADE) (https://gdt.gradepro.org/app/handbook/handbook.html) approach was used to assess quality or certainty of the evidence for the primary outcomes.^26^

### 2.8 Data synthesis

Narrative synthesis and meta‐analysis of the primary outcome variables were conducted. For adults we calculated the mean difference in weight and for children the standardized mean difference in BMI z‐score, both using the inverse variance method. We used a random effects model because we expected that interventions for the prevention of body weight gain would be heterogeneous in terms of content and implementation of the interventions, and, therefore, the true effect of the intervention is likely to vary between studies.^27^ For studies with more than one intervention group, data from all relevant treatment arms were included and the number of participants in the control arm was divided by the number of treatment arms.^28^ In addition, for studies with multiple follow‐up times, we used the immediate posttreatment measure as the main analysis of the effect. When selecting outcome data for pooling, we prioritized data that were adjusted for confounders, clustering, and data that were analyzed by intent‐to‐treat wherever possible. If the standard deviation was not reported or obtained from the study authors, when possible, we calculated it from the 95% confidence intervals, *p*‐values or SE, using the calculator provided in Review Manager 5.4.1 and equations available in Chapter 6 of the Cochrane Handbook 6.3.^19^

Heterogeneity was identified by visual inspection of forest plots, and the *I* ^*2*^ statistic, which quantifies inconsistencies in the studies, was also used to assess heterogeneity in the meta‐analysis. An *I* ^*2*^ statistic level of 75% or higher indicates a considerable level of heterogeneity.^21^ Where heterogeneity was found, we had planned to determine the possible reasons using subgroup analysis (festive holidays vs. non‐festive holidays, studies performed in winter vs. summer season, effect in people of different levels of adiposity, and effect on people of different ages), but this was not possible due to the low or null availability of studies in each subgroup. We also conducted sensitivity analysis (studies with a low risk of bias and fixed effects) for the studies in adults. We did not generate funnel plots to assess the presence of small‐study effects as there were less than 10 studies included for the primary outcomes. All statistical analyses were performed using Review Manager (RevMan, [Computer Program]. Version 5.4. The Cochrane Collaboration, 2020).

## 3 RESULTS

**FIGURE 1:**
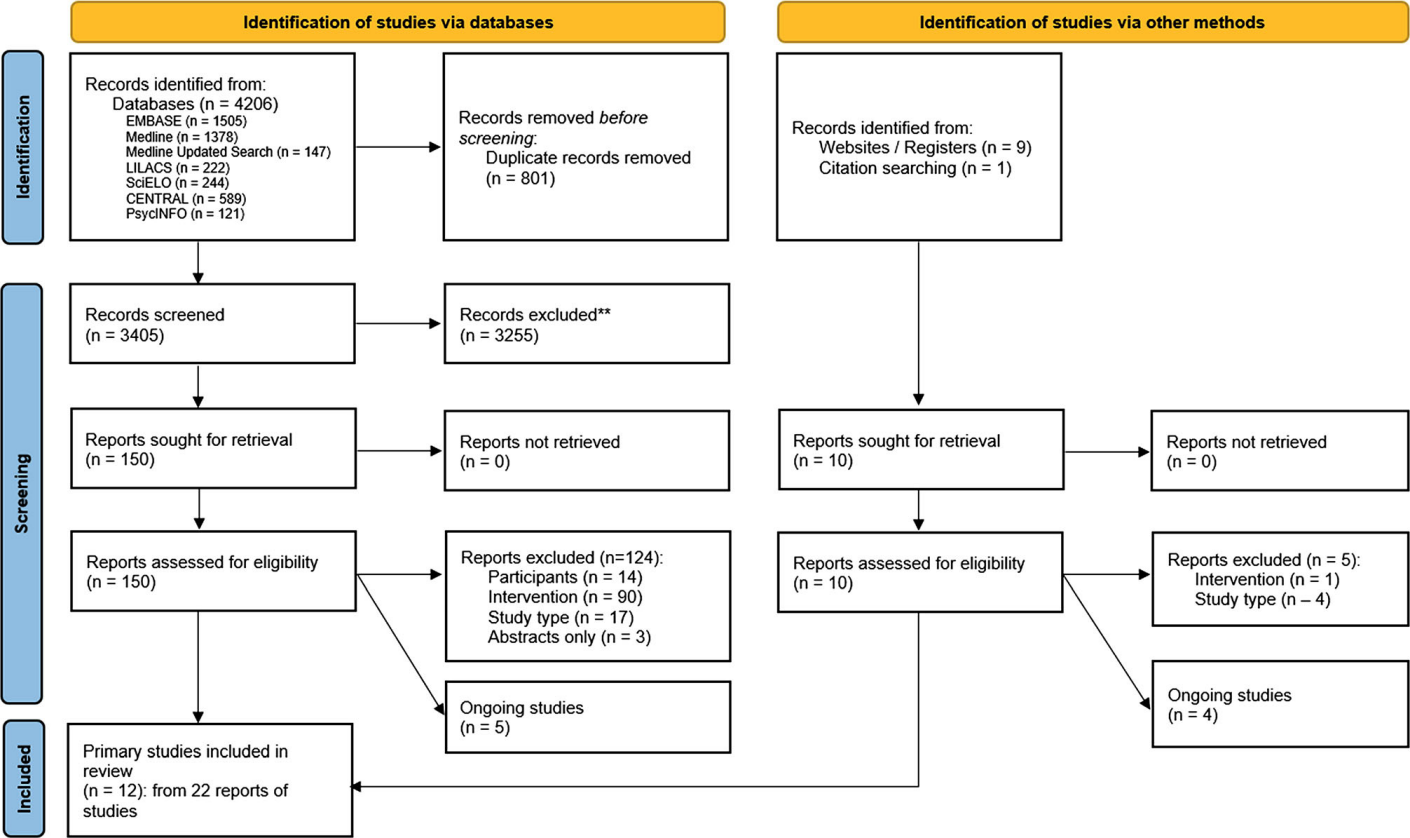
Study selection flow diagram for the systematic review of interventions to prevent weight gain in festive and holiday periods.

We identified 3415 records after removing duplicates (Figure 1 and Table S1). After screening of titles and abstracts and selection based on the full‐text articles, we included 22 reports, which represented 12 primary studies^17^, ^29^, ^30^, ^31^, ^32^, ^33^, ^34^, ^35^, ^36^, ^37^, ^38^, ^39^ with 10 supporting references.^40^, ^41^, ^42^, ^43^, ^44^, ^45^, ^46^, ^47^, ^48^, ^49^ An additional nine studies were identified as ongoing studies^50^, ^51^, ^52^, ^53^, ^54^, ^55^, ^56^, ^57^, ^58^ because only a conference abstract, study protocol, or trial registration was available (File S2). The most common reason for exclusion of the 129 reports at the full‐text stage was due to the intervention (*n* = 91), including that it was not implemented during the festive or holiday period, was aimed at weight reduction, or had a duration of less than 2 weeks, or a combination of these (Figure 1 and File S3).

### 3.1 Characteristics of included studies

A total of 12 primary studies were included. Eight RCTs,^17^, ^29^, ^31^, ^32^, ^33^, ^35^, ^37^, ^38^ one cluster‐RCT,^34^ and three non‐RCTs^30^, ^36^, ^39^ were found with a total of 670 children and adolescents (ages 6–13 years), and 489 adult participants included in the studies (Table 1). Eleven studies were conducted in the Northern hemisphere,^29^, ^30^, ^31^, ^33^, ^34^, ^35^, ^36^, ^37^, ^38^, ^39^ with 10 in the United States and one in the United Kingdom.^17^ One study was conducted in Chile,^32^ in the Southern hemisphere.

**TABLE 1:** Characteristics of included studies.

Study ID; country and references^a^	Study design; follow‐up from baseline	Participants—sample size; age range; sex (% male); race/ethnicity; income status	Intervention—description; interventionist; setting; use of theory	Outcomes measured
Children, Holiday—Summer break				
Baranowski, 2003; USA^30^	Two‐armed RCT12‐weeks	*N* = 35 girls and their parents or caregivers; 8‐year‐olds (mean 8.3 years); 0% male; African‐American; 46% had a household income <$40,000	Intervention group: GEMS‐FFFP special summer day camp, followed by a special home Internet intervention for the girls and their parents. Control group: summer day camp, followed by a monthly home Internet intervention without GEMS‐FFFP enhancements. Interventionist: N/R. Setting: Day camp. Use of theory: Social cognitive theory	Height, BW, BMI (kg/m^2^), FM%, WC
Evans, 2018; USA^31^, ^42^	Two‐armed non‐RCT8 weeks	*N* = 81; 6–12 years; 57.4% male; 18.25% non‐Hispanic White, 19.25% non‐Hispanic Black, 31.25% non‐Hispanic Other, 31.25% Hispanic (all races); Low income	Intervention group: Physical activity programming SPARK‐AS and lunch offered through the SFSP. Comparison group: SFSP open‐side at their housing community but no access to the intervention programming. Interventionist: College‐age summer staff. Setting: Community public park. Use of theory: Social cognitive theory	BMIz
Evans, 2020; USA^32^, ^43^	Two‐armed RCT7 or 8 weeks (2017, 2018)	*N* = 94; 6–12 years; 8.7% non‐Hispanic White, 15.25% non‐Hispanic Black, 13.5% non‐Hispanic other, 63% Hispanic (all races); Low income	Intervention group: Daily day camp offering physical activities including sports, arts and crafts. Free breakfast and lunch meals provided via the SFSP. Comparison group: Experienced summer vacation as planned by their parent/guardian without enrolling in summer day camp or other daily structured summer programming for more than 1 week. Interventionist: N/R. Setting: Day camp. Use of theory: N/R.	BMIz
Hopkins, 2019; USA^36^, ^49^, ^50^ ^,64^	Three‐armed cluster‐RCT8 weeks	N = 87; Kindergarten through 5th grade, Age range not reported (mean 7.56 years); 43.02% males89.53% Black, 10.47 non‐Black; Low income	Enhanced Care: Nutrition, physical activity, and mental health programming with access to free meals and safe play. Standard Care: Nutrition and physical activity programming with access to free meals and safe play. Active Control: access to free meals and safe play, (no nutrition, physical activity or mental health programming). Interventionist: N/R. Setting: Public schools. Use of theory: Social cognitive theory	BMIz
Kilanowski, 2015; USA^37^	Two‐armed non‐RCT12 weeks	*N* = 171; 55% males; 6–13 years; Race/Ethnicity: N/R; Income status: N/R	Intervention group: Nutrition and physical activity through calisthenics and sport lessons to migrant children. Control group: Received bilingual healthy eating, low‐literacy, publicly available CDC flyers on healthy eating. Interventionist: Part‐time media teacher, pediatric nurse practitioner. Setting: Midwest Migrant Education Program locations. Use of theory: N/R	BMI (kg/m^2^), BMIp
Meucci, 2013; USA^38^	Three‐armed RCT4‐ week or 8‐week	N = 22 adolescents; 55% males; 8–12 years; Race/Ethnicity: N/R; Income status: N/R	Intervention group: 4‐week or 8‐week group play‐based activity, nutrition classes and healthy snacks and lunches. Control group: Followed their usual summer break without any intervention from the study coordinators; however, they were asked to maintain their current level of physical activity for the duration of the study. Interventionist: Expert instructors. Setting: Day camp. Use of theory: Social cognitive theory	Height, BW, BMI (kg/m^2^), FM
von Klinggraeff, 2022; USA^40^, ^41^, ^50^	Three‐armed non‐RCT12‐weeks	*N* = 180; 7–9 years (mean 7.9 years); 40% males; 94% non‐Hispanic Black; Low‐income	HSL intervention: Alternated academic classes with physical activity, with 15‐min nutrition education session during lunch, plus healthy breakfast, lunch, and snack. 21C intervention: Academic sessions plus physical activity before lunch. Healthy breakfast and lunch. Control group: No program. Interventionist: N/R. Setting: School. Use of theory: Social cognitive theory	BMI (kg/m^2^)
Adults—Winter holiday (or Chilean National Holidays—Hernandez‐Jana 2010)				
Hernandez‐Jaña, 2020; Chile^33^	Two‐armed RCT3 weeks	*N* = 36; Age range not reported, (mean 20.91 years); Race/Ethnicity: N/R; Income status: N/R	Intervention group: Traditional nutritional session (lasted 20 min) that included a body composition measurement, nutritional assessment, and a brief educational talk about healthy eating. This group received healthy recommendations focused on the Chilean National Holidays. Control group: Were asked to continue their normal activities. Interventionist: N/R. Setting: University laboratory. Use of theory: N/R	BW, BMI (kg/m^2^), FM%.
Hirsh, 2019; USA^34^, ^44^	Two‐armed pilot RCT6‐weeks	*N* = 22; 21–45 years; 21.87% males; Race/Ethnicity: N/R; Income status: N/R	Intervention group: 52 days of intermittent energy restriction during 2 days per week (730 kcal/d; 3050 kJ/d) and 5 days of eating their habitual diet, with daily consumption of a set of dietary supplements. Control group: Followed their habitual diet without any restriction and daily intake of multivitamin for 52 days. Interventionist: N/R. Setting: Clinical setting. Use of theory: N/R	BW, AE
Kaviani, 2019; USA^36^, ^45^	Two‐armed RCT7 weeks	*N* = 111; 18–65 years; 26.12% males; Race/Ethnicity: N/R; Income status: N/R	Intervention group: Performed DSW. Participants were instructed to try not to gain weight, and no additional instructions on how to achieve that goal were provided. Control group: Did not receive any intervention. They completed the same study visits as the intervention group. Interventionist: N/R. Setting: Clinical setting. Use of theory: Social cognitive theory	BW, BMI (kg/m^2^), FM%, WC.
Mason, 2018; UK^18^, ^46^	Two‐armed RCT4–8 weeks	*N* = 272; 22% males; >18 years; white (78%); Income status: N/R	Intervention group: DSW, weight record and weight trajectory feedback; information of weight management strategies over the Christmas period; and PACE information for regularly consumed festive foods and drinks. The goal was to gain no more than 0.5 kg of baseline weight. Comparison group: received a leaflet on healthy living. Interventionist: N/R. Setting: home or convenient location. Use of theory: Self‐regulation theory and habit formation model	BW, FM%.
Watras, 2007; USA^39^	Two‐armed RCT6 months	*N* = 48 randomized, *N* = 40 completers; 18–44 years; 20% males; Race/Ethnicity: N/R; Income status: N/R	Intervention group: 4 g/day of 78% active CLA isomers of safflower oil (3.2 g/day CLA). Comparison group: 4 g/day of placebo (safflower oil). Interventionist: N/R. Setting: Clinical setting. Use of theory: None reported	BW, FM%, AE

Abbreviations: 21C, 21st Century Summer Learning Program; AE, Adverse Events; BMI, Body Mass Index; BMIz, Body Mass Index z‐score; BMIp, Body Mass Index percentiles; BW, body weight; CDC, Centers for Disease Control and Prevention; CLA, conjugated linoleic acid; DSW, daily self‐weighing; FM, fat mass kilograms; FM%, fat mass percentage; GEMS‐FFFP, girls health enrichment multisite studies‐fun, food, and fitness project; HSL, healthy summer learners; N/R, information not reported; PACE, physical activity calorie equivalents; RCT, randomized controlled trial; SFSP, summer food service program; SPARK‐AS, Sports, Play Active Recreation for Kids After School; UK, United Kingdom; USA, United States of America; WC, waist circumference.

^a^ For studies with more than one reference listed, the first is the primary reference.

All the included studies with child and adolescent participants had a focus on weight gain prevention during the summer school holidays (*n* = 7).^29^, ^30^, ^31^, ^34^, ^36^, ^37^, ^39^ For the five studies with adult participants, four were focused on the prevention of weight gain during the December holiday season,^17^, ^33^, ^35^, ^38^ and one was aimed at weight gain prevention during the Chilean national holidays,^32^ which last for between 7 and 9 days in September.

### 3.2 Intervention

Brief information about the interventions is in Table 1, with further details in Table S2. Seven interventions that were included in this systematic review used cognitive behavioral theory.^17^, ^29^, ^30^, ^34^, ^35^, ^37^, ^39^ This theory was applied by using self‐regulatory strategies with participants that involved self‐efficacy. They usually suggested to participants that they must state goals to achieve changes in their behavior that are meant to prevent increases in BMI z‐score/percentiles (for children and adolescents) or weight gain (for adults).

The interventions lasted from 4 to 12 weeks in children and adolescents. For adults, the interventions ranged from one nutritional counselling session prior to the holiday period^32^ up to 6 months of conjugated linoleic acid (CLA) supplementation.^38^ Follow‐up measurements were taken at the end of the holiday period for all studies. Two studies also reported measures after the end of the holiday period, Kaviani et al.^35^ also took measures 14 weeks after the end of the holiday period, and von Klinggraeff et al.^39^ also took measures 12‐months from baseline. The majority of the studies implemented in children and adolescents were delivered face‐to‐face during day camp visits. Interventions delivered to adults were delivered face‐to‐face in university laboratories and included strategies such as reading leaflets and self‐weighing, nutrition classes, and taking daily doses CLA in capsules.

### 3.3 Risk of bias assessment in included studies

Of the eight RCTs evaluated with the RoB 2 tool (three studies in children and adolescents, and five in adults), six were classified as having a high risk of bias^29^, ^31^, ^32^, ^33^, ^37^, ^38^ and two as low risk of bias^17^, ^35^ (Figure S1a). Bias due to “deviations from the intended interventions” (domain 2), “missing outcome data” (domain 3), and “measurement of the outcome” (domain 4) were the most serious issues influencing the RoB 2 assessment. Only one study in children was evaluated with ROB 2 for cluster‐RCT^34^ (Figure S1b), having a high risk of bias overall. The main issue with this study was related to “measurement of the outcome” (domain 4). Three non‐RCTs in children and adolescents^30^, ^36^, ^39^ were assessed with the ROBINS‐I tool (Figure S1c), with two having a moderate and one a critical overall risk of bias results. The main problems were related to “bias due to confounding” and “bias due to missing data.”

For the studies in children and adolescents, the main risk of bias concerns were related to “missing outcome data,” “measurement of the outcome,” “confounders,” and “randomization process.” These problems were related to a lack of explanation by the authors of the reasons for participants deserting the study being lost to follow‐up, lack of information about the outcome assessor (and whether they were blinded to intervention group), and the absence of a multiple regression model to control for confounders in the analysis (for studies without randomization). For the four studies that did use randomization, authors of three studies did not report the randomization process (e.g., simple, block, and stratified).

For the studies in adults, the main concerns were related to “deviations from intended interventions” and “measurement of the outcome.” This was due to a lack of information about whether the participants and outcome assessors were blinded to the intervention group. Some concerns were related to the “randomization process” and “selection of reported result” due to a failure to report the allocation concealment process and statistical analysis plan, respectively.

### 3.4 Effects of interventions

**FIGURE 2:**
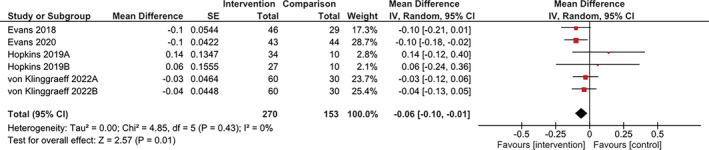
Meta‐analysis of studies implemented in child and adolescent populations to prevent increases in BMIz‐score units during the summer school holiday period. Note: For the Hopkins 2019 study, A and B refer to the enhanced care and standard care interventions, respectively. For the von Klinggraeff 2022 study, A and B refer to the healthy summer learners and 21st century summer learning program interventions, respectively.

Key findings of the included studies can be found in Table S3. Of the seven studies in children and adolescents, only one study showed a significant effect of the intervention on the primary outcome measure.^31^ For the other six studies, three showed a positive trend favoring the intervention,^30^, ^36^, ^39^ two showed a negative trend,^29^, ^34^ and one showed no effect.^37^ A meta‐analysis of four studies with suitable outcome data,^30^, ^31^, ^34^, ^39^ which represented six intervention arms, showed a significant overall effect on BMI z‐score of −0.06 (95% CI [−0.10, −0.01] kg, *p* = 0.01; four studies, 423 participants [270 intervention group and 153 control group]; *I* ^2^ = 0%) favoring the intervention group (Figure 2). The overall quality of evidence for interventions to prevent increase in BMI z‐score in children during summer was very low (Table S4) due to limitations in the high risk of bias assessment, inconsistency of the results and design of the included studies. It is important to keep in mind that due to the failure to obtain data suitable for meta‐analysis from three of the seven studies (representing 43% of all participants), the results from this analysis may not be representative of all included studies. Also, the moderate risk of bias of two studies^30^, ^39^ and high risk of bias of the other two studies^31^, ^34^ included in the meta‐analysis suggest the results should be taken with caution. Only one study had a longer term follow‐up, showing a small, nonsignificant increase in the difference between groups at 12‐months of follow‐up^39^ (Table S3).

**FIGURE 3:**
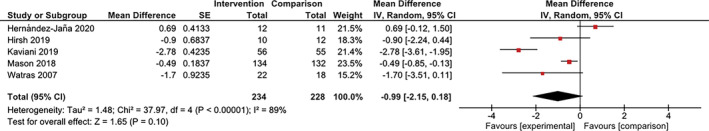
Meta‐analysis of studies in adults to prevent increases in body weight during festive periods.

Of the five studies in adults, four reported the difference in change in weight as an outcome after an intervention applied during the December holiday period.^17^, ^33^, ^35^, ^38^ Three found a significant positive effect of the intervention^17^, ^35^, ^38^ and one found a nonsignificant but positive trend favoring the intervention.^33^ The fifth study reported the difference in change in fat mass as an outcome after an intervention applied during the Chilean national holidays.^32^ Through contact with the corresponding author, we obtained data on body weight at follow‐up, which showed a nonsignificant negative effect of the intervention. A meta‐analysis of these five studies^17^, ^32^, ^33^, ^35^, ^38^ showed an overall effect on body weight of −0.99 kg (95% CI [−2.15, 0.18], *p* = 0.10; five studies, 462 participants [234 intervention group and 228 control group]; *I* ^2^ = 89%) (Figure 3). These results should be treated with caution due to the high heterogeneity and the high risk of bias in three of the studies.^32^, ^33^, ^38^ The overall quality of evidence for interventions to prevent increases in body weight gain during festive periods in adults was very low (Table S5). Limitations impacting on the overall quality of evidence were the high risk of bias of studies, inconsistency of the results, and differences in population and interventions that had an impact on indirectness and imprecision of the effect. Only one study had a longer term follow‐up, which was 21 weeks from baseline, that showed a reduction in the benefit^35^ (Table S3). The sensitivity analysis for fixed effects (vs. random effects) showed a smaller but significant effect of the intervention of −0.67 kg (95% CI [−0.96, −0.38], *p* < 0.00001; *I* ^2^ = 89%) (Figure S2). And, for the two studies with a low risk of bias the effect of the intervention was −1.60 kg (95% CI [−3.85, 0.64], *p* = 0.16; *I* ^2^ = 96%) (Figure S3).

Though not previously specified, we also conducted a subgroup analysis for type of holiday period (December holiday period vs. Chilean national holidays) as a possible explanation for the heterogeneity (Figure S4). This showed a significant overall effect of the interventions in the December holiday period of −1.45 kg (95% CI [−2.78, −0.11], *p* = 0.03; four studies, 439 participants [222 intervention group and 217 control group]; *I* ^2^ = 88%) with moderate heterogeneity.

Only two of the 12 included studies reported having measured adverse effects of the interventions. Ten mild to moderate adverse effects, possibly related to the dietary intervention (intermittent fasting), were reported by Hirsh, et al.^33^ Reported adverse effects were gastrointestinal (flatulence and nausea) and intermittent, with no treatment required. Watras et al.^38^ reported a significantly different from placebo decrease in frequency of reported emotional symptoms (*p* = 0.04) in the CLA supplement intervention group.

## 4 DISCUSSION

Given the evidence that shows that some periods during the year, such as festive and summer holiday periods, are associated with weight gain in adults and children, respectively, interventions are needed to prevent weight gain in these critical periods. The present systematic review was conducted to determine which interventions have been tested and whether they are effective. For children, the effect of the interventions on BMI z‐score was positive overall (−0.06, 95%CI −0.10, −0.01).^30^, ^31^, ^34^, ^39^ However, these results should be taken with caution due to the risk of bias, inconsistency in the results and the very low quality of evidence. For adults, the evaluated interventions showed a consistent positive effect on prevention of weight gain during the December holiday period compared to a control group (−1.45 kg, 95%CI −2.78, −0.11), but not during the Chilean national holidays (0.69 kg, 95%CI −0.12, 1.5).^17^, ^32^, ^33^, ^35^, ^38^ The high risk of bias of three of the studies, high heterogeneity, and the very low quality of the evidence suggests that these results should be taken with caution. The effect in adults remained positive under the different sensitivity analyses.

To our knowledge, this is the first systematic review that has evaluated the risk of bias, quality of the evidence and reports the effects of interventions to prevent weight gain in adults, and BMI z‐score/percentiles in children and adolescents during festive and/or holiday periods. The interventions in children and adolescents, all conducted in the United States, showed a positive trend toward preventing BMI increases during the summer season. These interventions included the self‐efficacy principle as the main tool to achieve this, in combination with physical activity and an intervention duration from 6 to 12 weeks. However, the lack of reporting of suitable outcome data for meta‐analysis for three of the studies, and limitations with the study methods and reporting, that increased the risk of bias and decreased the quality of evidence of most of the studies prevent firm conclusions. The change in BMI z‐score found in our review was higher than that found in a recently published Cochrane systematic review of interventions in any setting to prevent obesity in children aged 5 to 11 years.^59^ They found an overall effect on BMI z‐score of dietary and physical activity interventions of −0.03, (95% CI [−0.06, 0.00], 26 studies, 12,784 participants) with short‐term interventions (12 weeks to <9 months). In contrast to our review, most of the studies were conducted during school time and had a minimum of 12 weeks of follow‐up. The intervention duration and follow‐up time was generally shorter for the studies included in our review. Like our review, there were limitations in how the studies were done, not all studies reported results in a way that they could be included in a meta‐analysis, and most studies were conducted in high‐income countries.^59^

The evidence for adolescents is limited, as the maximum age of the children included in the studies was 13 years, and the results may not be generalizable to countries outside of the United States. Unlike our review, a recent Cochrane systematic review of interventions in any setting to prevent obesity in adolescents found 74 studies for this age group, of which 54 could be included in a meta‐analysis.^60^ The review found that strategies to encourage adolescents to change their diet or activity levels (or both) made no or very little difference to their BMI. Most studies were conducted in high‐income countries, in the school setting, and had limitations in the way the studies were done.

In adults, interventions to prevent weight gain during the December holiday season were the most promising, including two studies with a low risk of bias that each had significant results. However, the studies were all conducted in the United States or United Kingdom so may not be generalizable to other countries, and the high heterogeneity suggests that these results should be taken with caution. Most of the interventions included improving self‐efficacy as a cognitive behavioral technique. The focus of the interventions was on intermittent fasting for two continuous days, self‐weighing and/or in being conscious of overeating during this critical period.^17^, ^33^, ^35^ They had a duration of 7 to 8 weeks. Also, the use of CLA could be an additional approach with benefits during this period, but the evidence is limited to one study with a high risk of bias.^38^ The negative effect of the intervention during the Chilean national holidays^32^ could be due to any of several reasons, including the low intensity of the intervention, the shorter holiday period, and the high risk of bias of the study. The difference in weight between intervention and control groups of −0.99 kg (95% CI −2.15, 0.18) found in our review for all studies, and −1.45 kg (95%CI −2.78, −0.11) for studies in the December holiday period is similar to the result found in a systematic review published in 2021 of weight gain prevention interventions in adults by Martin et al.^61^ Their meta‐analysis of 29 studies found a difference of −1.15 kg (95%CI −1.50, −0.80). Unlike our review, the authors only included studies in adults aged 18–50 years, published in English and that tested lifestyle interventions (diet and/or physical activity). Thus, only the study by Mason et al.^17^ that was included in our review was also included in the review by Martin et al. Given the larger number of included studies, they were able to conduct subgroup analyses that showed greater effects in nonobese populations and greater efficacy with prescriptive than nonprescriptive lifestyle interventions. Prescriptive interventions were characterized by pre‐specified, structured dietary and/or physical activity targets.^61^

Strengths of this review are that we have a prior registration of the systematic review protocol, our PICOS criteria were clearly established, and we included gray and published literature with no date or language limits. Further, study selection, data extraction, and risk of bias assessment were performed in duplicate, and the search strategy was comprehensive. Limitations of the review are that we have not been able to include in the meta‐analysis all seven studies in the child and adolescent population due to lack of suitable outcome data. Further the four studies that were included had moderate or high risk of bias. While three of the five included RCTs in adults had a high risk of bias, the two RCTs with low risk of bias both showed positive effects of the intervention on weight gain prevention −1.60 kg (95% CI [−3.85, 0.64], *p* = 0.16; *I* ^2^ = 96%).^17^, ^35^ We could not assess the risk of publication bias due to the lack of sufficient studies for funnel plots. However, we expect that our comprehensive search strategy, including clinical trial registries, minimized the possibility of publication bias. Another limitation of this work is that we could not determine if the benefit from the interventions is lost in the long‐term. Only two studies had longer term follow‐up measures, with one showing a reduction in the effect of the intervention, and the other a small, nonsignificant increase.^35^, ^39^

### 4.1 Implications for policy and practice

Evidence in this systematic review shows that a promising strategy, with no reported harms or adverse effects, that could be implemented in children is an intervention composed of a daily day camp during the summer holiday period, offering a diverse range of physical activities such as sports and arts and crafts, complemented by nutritious, free breakfast and lunch. The intervention should have a duration between 6 to 8 weeks for 7 to 8 h daily from Monday to Friday. Ideally, this intervention would be an extension of similar activities conducted in school time to ensure success. In countries outside of the United States, especially, it is recommended that this program be delivered as part of a rigorous, high‐quality RCT or cluster‐RCT. Such an approach will further contribute to the evidence base and enhance our understanding of its effects for children.

For adults, a promising intervention involves daily self‐weighing combined with nutrition counselling.^17^, ^35^ In these two RCTs with a low risk of bias, the interventions applied were based on social cognitive theory with a focus on self‐efficacy and applied using a personal feedback cycle and establishment of logical and achievable goals for the individual for physical activity and healthy eating behaviors. Participants were recommended to weigh themselves at least twice a week, but preferably daily, to allow self‐monitoring of their weight. The intervention by Mason et al.^17^ also used brochures of energy equivalents in calories to minutes of physical activity using the energy contained in common foods during festive periods in adults as well as simple healthy eating tips (e.g., do not consume food in a hurry, keep the consumption of ultra‐processed foods to a minimum). The duration of the intervention should be at least 4 to 8 weeks and should include the period from mid‐ to late‐November to early January. To build on the evidence base, it is crucial to test these interventions as part of high‐quality RCTs in countries outside of the United States and United Kingdom. This will provide valuable insights into their effectiveness and applicability across diverse populations.

### 4.2 Implications for research

An important finding of this systematic review is that there is a need for better quality studies in both children and adults. For studies in children and adolescents, authors should report the reasons for participants being lost to follow‐up, clearly state whether the outcome assessor is blinded to the intervention group, use a multiple regression model to control for confounders in the analysis (for studies that do not use randomization), and report the randomization process for RCTs. For the studies in adults, authors should clearly report whether the participants and outcome assessors were blinded to the intervention group, detail the allocation concealment process, and report their pre‐specified statistical analysis plan.

Interventions of higher intensity and longer duration (see suggestions in the previous section) are needed to improve results in children and adults, and with larger sample sizes. Research gaps include the adolescent age group and, for all age groups, studies outside of the United States and the United Kingdom, including in low‐ and middle‐income countries and in other cultural groups.

## 5 CONCLUSION

The evidence for interventions targeting prevention of BMI increases in children during summer showed positive results, while interventions to prevent weight gain in adults during December holiday periods also showed consistent positive effects. Evidence with a low risk of bias is sparse in both populations. Studies with better quality design, larger sample size, higher intensity, and longer duration and performed with target populations outside the United States are needed to improve results in children and adults. Finally, there is a relative gap in the evaluation of interventions to prevent BMI increases during the summer break in adolescents. This review has highlighted potential interventions to prevent the increase in body weight during the festive and holiday periods, particularly in the United States and United Kingdom. However, more work is needed to further improve the quality of the evidence and to extend it to other countries and to the adolescent population.

## AUTHORS CONTRIBUTIONS

Diego E. Guerrero‐Magaña, Michelle M. Haby, and Rolando G. Díaz‐Zavala conceptualized and designed the protocol. Diego E. Guerrero‐Magaña drafted the initial manuscript, with input from Michelle M. Haby. Diego E. Guerrero‐Magaña and Michelle M. Haby defined the concepts and search items, data extraction process, and methodological appraisal of the included studies. Diego E. Guerrero‐Magaña and Lucía G. Urquijo‐Ruiz screened the titles and abstracts of potential records. Diego E. Guerrero‐Magaña, Lucía G. Urquijo‐Ruiz, Alma L. Ruelas‐Yanes, Teresita de J. Martínez‐Contreras, and Michelle M. Haby applied the inclusion criteria to the full‐text articles. Data extraction and risk of bias assessment were performed by Diego E. Guerrero‐Magaña, Alma L. Ruelas‐Yanes, and Michelle M. Haby. Michelle M. Haby, Rolando G. Díaz‐Zavala, Maria del Carmen Candia‐Plata, and Julián Esparza‐Romero contributed with their expertise in systematic review conduct and topic knowledge. All authors provided conceptual input and revised and approved the final version of the manuscript.

## CONFLICT OF INTEREST STATEMENT

The authors declare that there is no support from any organization for the work presented; no financial relationship with any person who may have an interest in the work submitted in the previous 3 years; and there are no other relationships or activities that have influenced the work submitted.

## Supporting information


**File S1** Search strategy for each database.
EMBASE search strategy
MEDLINE search strategy
CENTRAL search strategy
PsycINFO search strategy
SciELO search strategy
LILACS search strategy
Updated MEDLINE Search
**Table S1.** Table of search results by database and supplementary search.
**File S2.** List of ongoing studies (n = 9).
**File S3.** List of excluded studies (n=129), with reason for exclusion.
**Table S2.** Intervention details for included studies.
**Figure S1.** Risk of bias assessment in included studies.
**Table S3.** Key findings of included studies.
**Figure S2**. Sensitivity analysis of studies in adults – fixed effects model.
**Figure S3**. Sensitivity analysis of studies in adults – low risk of bias studies.
**Figure S4.** Subgroup analysis for type of holiday period for studies in adults.
December holiday period vs Chilean national holidays.
**Table S4.** GRADE assessment of quality of evidence for studies in children and adolescents.
**Table S5.** GRADE assessment of quality of evidence for studies in adults.
