# Supplementary material for: Interventions for the prevention of weight gain during festive and holiday periods in children and adults: A systematic review
Source: Obes Rev. 2024 Sep 14;26(1):e13836. doi: 10.1111/obr.13836 (PMC11611437; doi:10.1111/obr.13836)
Supplement: Supplementary file 1 — File S1 Search strategy for each database. EMBASE search strategy MEDLINE search strategy CENTRAL search strategy PsycINFO search strategy SciELO search strategy LILACS search strategy Updated MEDLINE Search Table S1. Table of search results by database and supplementary search. File S2. List of ongoing studies (n = 9). File S3. List of excluded studies (n=129), with reason for exclusion. Table S2. Intervention details for included studies. Figure S1. Risk of bias assessment in included studies. Table S3. Key findings of included studies. Figure S2. Sensitivity analysis of studies in adults – fixed effects model. Figure S3. Sensitivity analysis of studies in adults – low risk of bias studies. Figure S4. Subgroup analysis for type of holiday period for studies in adults. December holiday period vs Chilean national holidays. Table S4. GRADE assessment of quality of evidence for studies in children and adolescents. Table S5. GRADE assessment of quality of evidence for studies in adults. [file OBR-26-e13836-s001.docx]

**SUPPLEMENTARY MATERIAL**

INTERVENTIONS FOR THE PREVENTION OF WEIGHT GAIN DURING FESTIVE AND HOLIDAY PERIODS IN CHILDREN AND ADULTS: A SYSTEMATIC REVIEW

**Authors and institutions:**

Diego E. Guerrero-Magaña^1,2^, MSPH. E-mail: [diego.guerrero@unison.mx](mailto:diego.guerrero@unison.mx)

Lucía G. Urquijo-Ruiz^1^, BS. E-mail: [a213216685@unison.mx](mailto:a213216685@unison.mx)

Alma L. Ruelas-Yanes^1^, MSPH. E-mail: [a211209573@unison.mx](mailto:a211209573@unison.mx)

Teresita de J. Martínez-Contreras^2^, MSPH. E-mail: [teresita.martinez@unison.mx](mailto:teresita.martinez@unison.mx)

Rolando G. Díaz-Zavala^2^, PhD. E-mail: [giovanni.diaz@unison.mx](mailto:giovanni.diaz@unison.mx)

Maria del Carmen Candia-Plata^3^, PhD. E-mail: [carmen.candia@unison.mx](mailto:carmen.candia@unison.mx)

Julián Esparza-Romero^4^, PhD. E-mail: [julian@ciad.mx](mailto:julian@ciad.mx)

Michelle M. Haby^2,5^, PhD. E-mail: [haby@unimelb.edu.au](mailto:haby@unimelb.edu.au)

^1^ Posgrado en Ciencias de la Salud, Facultad Interdisciplinaria de Ciencias Biológicas y de la Salud, Universidad de Sonora, Blvd. Luis Encinas y Rosales S / N, C.P. 83000. Hermosillo, Sonora, México.

^2^ Departamento de Ciencias Químico-Biológicas, Facultad Interdisciplinaria de Ciencias Biológicas y de la Salud, Universidad de Sonora, Blvd. Luis Encinas y Rosales S / N, C.P. 83000. Hermosillo, Sonora, México.

^3^ Departamento de Medicina y Ciencias de la Salud, Facultad Interdisciplinaria de Ciencias Biológicas y de la Salud, Universidad de Sonora, Blvd. Luis Encinas y Rosales S / N, C.P. 83000. Hermosillo, Sonora, México.

^4^Unidad de Investigación en Obesidad y Diabetes, Coordinación de Nutrición, Centro de Investigación en Alimentación y Desarrollo (CIAD), Carretera Gustavo Enrique Astiazarán Rosas, No.46. Col. La Victoria, C.P.83304, Hermosillo, Sonora

^5^Melbourne School of Population and Global Health, The University of Melbourne, Parkville, Victoria, 3010 Australia.

**CONTENTS**

| [**File S1.** Search strategy for each database](#_Supplementary_file_1.)  [EMBASE search strategy](#_EMBASE_search_strategy:)  [MEDLINE search strategy](#_MEDLINE_search_strategy:)  [CENTRAL search strategy](#_CENTRAL_search_strategy:)  [PsycINFO search strategy](#_PsycINFO_search_strategy:)  [SciELO search strategy](#_SciELO_search_strategy:)  [LILACS search strategy](#_LILACS_search_strategy:)  [Updated MEDLINE Search](#_Updated_MEDLINE_Search) | 3  3  6  8  10  13  14  15 |
| --- | --- |
| [**Table S1.** Table of search results by database and supplementary search](#_Supplementary_file_2.) | 18 |
| [**File S2.** List of ongoing studies (n = 9)](#_Supplementary_file_3.) | 19 |
| [**File S3.** List of excluded studies (n=129)](#_Supplementary_file_4.), with reason for exclusion | 20 |
| [**Table S2.** Intervention details for included studies](#_Supplementary_file_5.) | 35 |
| [**Figure S1.** Risk of bias assessment in included studies](#_Supplementary_file_6.) | 38 |
| [**Table S3.** Key findings of included studies](#_Table_S3._Key) | 39 |
| [Figure S2. Sensitivity analysis of studies in adults – fixed effects model](#_Figure_S1._Sensitivity_1) | 42 |
| [Figure S3. Sensitivity analysis of studies in adults – low risk of bias studies](#_Figure_S2._Sensitivity_1) | 43 |
| **[Figure S4.](#_Figure_S3._Subgroup)** [Subgroup analysis for type of holiday period for studies in adults –](#_Figure_S3._Subgroup)  [December holiday period vs Chilean national holidays](#_Figure_S3._Subgroup) | 44 |
| [**Table S4.** GRADE assessment of quality of evidence for studies in children and adolescents](#_Table_S3._GRADE) | 45 |
| [**Table S5.** GRADE assessment of quality of evidence for studies in adults](#_Table_S4._GRADE) | 46 |
|  |  |

# File S1. Search strategy for each database

# EMBASE search strategy:

Database: Embase Classic+Embase <1947 to 2021 Week 10>

Search Strategy:

--------------------------------------------------------------------------------

1 cognitive behavioral therapy/ (14625)

2 dietary supplement/ (14942)

3 fasting/ (11884)

4 obesity/pc [Prevention] (13264)

5 short term psychotherapy/ (328)

6 social support/ (96562)

7 primary prevention/ (42401)

8 ((psychological or behavio?r$) adj (therapy or modif$ or strateg$ or intervention$)).tw,kw. (68292)

9 (group therapy or family therapy or cognitive therapy).tw,kw. (18019)

10 ((lifestyle or life style) adj (chang$ or intervention$)).tw,kw. (24922)

11 counsel?ing.tw,kw. (147375)

12 social support.tw,kw. (53673)

13 (peer adj2 support).tw,kw. (7739)

14 (diet$ adj (modif$ or therapy or intervention$ or strateg$)).tw,kw. (26075)

15 (low calorie or calorie control$ or healthy eating).tw,kw. (15735)

16 (fasting or modified fast$).tw,kw. (179883)

17 (fruit$ or vegetable$).tw,kw. (177963)

18 (high fat$ or low fat$ or fatty food$).tw,kw. (81307)

19 (aerobics or physical therapy or physical activity or physical inactivity or exercis$).tw,kw. (597501)

20 (fitness adj (class$ or regime$ or program$)).tw,kw. (1286)

21 (physical training or physical education or dance therapy or sendentary behavio?r).tw,kw. (16456)

22 (weightwatcher$ or weight watcher$).tw,kw. (228)

23 (fat camp$ or diet$ camp$).tw,kw. (30)

24 (health promotion or health education).tw,kw. (82352)

25 (media intervention$ or community intervention$).tw,kw. (3298)

26 (family intervention$ or parent$ intervention or health promoting school$).tw,kw. (3663)

27 ((school or community) adj2 (program$ or intervention$)).tw,kw. (31776)

28 (parent$ adj2 (behavio?r or involve$ or control$ or attitude$ or educat$)).tw,kw. (32491)

29 (health polic$ or school polic$ or food polic$ or nutrition polic$).tw,kw. (43671)

30 (primary prevention or secondary prevention or preventive measure$ or preventative measure$).tw,kw. (95555)

31 (weight gain adj2 prevention).tw,kw. (710)

32 (self-weigh* or nutrition or energy restrict$ or program$ or energy reduc$).tw,kw. (1485661)

33 or/1-32 (2838287)

34 (feast* or celebrat* or banquet* or holiday* or vacation* or "special occasion*" or "special event*" or thanksgiving or christmas or easter or ramadan or festiv* or diwali or religio* or birthday* or eid or dussera or "Ganesha Chaturthi" or Holi or "Krishna Janmashtami" or "Maha Shivratri" or Navratri or "Raksha Bandhan" or uposatha or "makha bucha" or "makha puja" or vesak* or "asalha puja" or dhamma or kathina or sanghamitta or "lantern festival" or qingming or "dragon boat" or "double seventh" or "autumn festival" or "double ninth" or "winter solstice" or laba).tw,kw. (93525)

35 (birthday* or wedding* or marriage* or "new year*" or hanukkah or chanukah or passover or Shabbat or "rosh chodesh" or "rosh hashanah" or "tzom gedalia" or "yom kippur" or sukkot or "simchat torah" or purim or shavuot).tw,kw. (40355)

36 (season* or summer or winter or autumn or winter or school holiday*).tw,kw. (276387)

37 ("fiestas decembrinas" or fiesta or navidad or semana santa or pascua).tw,kw. (786)

38 (holiday or vacation).tw,kw. (7400)

39 season/ (81123)

40 or/34-39 (416215)

41 Randomized controlled trial/ (653781)

42 Controlled clinical study/ (467189)

43 random$.ti,ab. (1659991)

44 randomization/ (90840)

45 intermethod comparison/ (270000)

46 placebo.ti,ab. (326948)

47 (compare or compared or comparison).ti. (565438)

48 ((evaluated or evaluate or evaluating or assessed or assess) and (compare or compared or comparing or comparison)).ab. (2284122)

49 (open adj label).ti,ab. (86212)

50 ((double or single or doubly or singly) adj (blind or blinded or blindly)).ti,ab. (248781)

51 double blind procedure/ (185239)

52 parallel group$1.ti,ab. (27248)

53 (crossover or cross over).ti,ab. (111655)

54 ((assign$ or match or matched or allocation) adj5 (alternate or group$1 or intervention$1 or patient$1 or subject$1 or participant$1)).ti,ab. (353868)

55 (assigned or allocated).ti,ab. (416863)

56 (controlled adj7 (study or design or trial)).ti,ab. (378294)

57 (volunteer or volunteers).ti,ab. (262566)

58 human experiment/ (540075)

59 trial.ti. (333151)

60 or/41-59 (5422781)

61 random* adj sampl* adj7 (“cross section$” or questionnaire$1 or survey* or database$1)).ti,ab. not (comparative study/ or controlled study/ or randomised controlled.ti,ab. or randomized controlled.ti,ab. or randomly assigned.ti,ab.) {Including Related Terms} (552)

62 Cross-sectional study/ not (randomized controlled trial/ or controlled clinical study/ or controlled study/ or randomised controlled.ti,ab. or randomized controlled.ti,ab. or control group$1.ti,ab.) {Including Related Terms} (10164)

63 (((case adj control$) and random$) not randomi?ed controlled).ti,ab. (18337)

64 (Systematic review not (trial or study)).ti. (169410)

65 (nonrandom$ not random$).ti,ab. (16982)

66 "Random field$".ti,ab. (2491)

67 (random cluster adj3 sampl$).ti,ab. (1351)

68 (review.ab. and review.pt.) not trial.ti. (875622)

69 "we searched".ab. and (review.ti. or review.pt.) {Including Related Terms} (0)

70 "update review".ab. (113)

71 (databases adj4 searched).ab. (41449)

72 (rat or rats or mouse or mice or swine or porcine or murine or sheep or lambs or pigs or piglets or rabbit or rabbits or cat or cats or dog or dogs or cattle or bovine or monkey or monkeys or trout or marmoset$1).ti. and animal experiment/ (1105904)

73 Animal experiment/ not (human experiment/ or human/) {Including Related Terms} (61891)

74 or/61-73 (2183550)

75 60 not 74 (5030467)

76 body weight loss/ (46885)

77 body weight gain/ (20917)

78 body weight/ (348375)

79 body mass/ (476305)

80 waist hip ratio/ (15370)

81 adipose tissue/ (90010)

82 body fat/ (42557)

83 (weight gain or weight loss).tw,kw. (236865)

84 (overweight or over weight or overeat$ or over eat$).tw,kw. (118078)

85 weight change$.tw,kw. (17879)

86 ((bmi or body mass index or zbmi or weight) adj2 (gain or loss or change or increase or decrease or lower or higher)).tw,kw. (332511)

87 or/76-86 (1132874)

88 33 and 40 and 75 and 87 (1505)

***************************

# MEDLINE search strategy:

Database: Ovid MEDLINE(R) and Epub Ahead of Print, In-Process, In-Data-Review & Other Non-Indexed Citations, Daily and Versions(R) <1946 to March 11, 2021>

Search Strategy:

--------------------------------------------------------------------------------

1 Cognitive Behavioral Therapy/mt [Methods] (13640)

2 Dietary Supplements/ (60488)

3 Fasting/ (35768)

4 Overweight/pc [Prevention & Control] (2072)

5 Obesity/dh, pc [Diet Therapy, Prevention & Control] (23511)

6 exp Psychotherapy, Brief/mt [Methods] (1657)

7 Behavior Therapy/mt [Methods] (11366)

8 Self Care/mt [Methods] (8200)

9 Social Support/ (72652)

10 Exercise/ (115593)

11 Health Policy/ (67994)

12 Primary Prevention/mt [Methods] (6763)

13 Health Promotion/ (75625)

14 Health Education/ (61437)

15 ((psychological or behavio?r$) adj (therapy or modif$ or strateg$ or intervention$)).af. (86500)

16 (group therapy or family therapy or cognitive therapy).af. (18722)

17 ((lifestyle or life style) adj (chang$ or intervention$)).af. (16801)

18 counsel?ing.af. (143140)

19 social support.af. (93316)

20 (peer adj2 support).af. (5554)

21 (diet$ adj (modif$ or therapy or intervention$ or strateg$)).af. (33138)

22 (low calorie or calorie control$ or healthy eating).af. (11384)

23 (fasting or modified fast$).af. (126715)

24 (fruit$ or vegetable$).af. (178778)

25 (high fat$ or low fat$ or fatty food$).af. (56122)

26 (aerobics or physical therapy or physical activity or physical inactivity or exercis$).af. (580943)

27 (fitness adj (class$ or regime$ or program$)).af. (973)

28 (physical training or physical education or dance therapy or sendentary behavio?r).af. (50287)

29 (weightwatcher$ or weight watcher$).af. (221)

30 (fat camp$ or diet$ camp$).af. (26)

31 (health promotion or health education).af. (219284)

32 (media intervention$ or community intervention$).af. (2637)

33 (family intervention$ or parent$ intervention or health promoting school$).af. (2791)

34 ((school or community) adj2 (program$ or intervention$)).af. (39493)

35 (parent$ adj2 (behavio?r or involve$ or control$ or attitude$ or educat$)).af. (40205)

36 (health polic$ or school polic$ or food polic$ or nutrition polic$).af. (162916)

37 (primary prevention or secondary prevention or preventive measure$ or preventative measure$).af. (94359)

38 (weight gain adj2 prevention).af. (570)

39 (self-weigh* or nutrition or energy restrict$ or program$ or energy reduc$).af. (2023870)

40 or/1-39 (3355953)

41 (feast* or celebrat* or banquet* or holiday* or vacation* or "special occasion*" or "special event*" or thanksgiving or christmas or easter or ramadan or festiv* or diwali or religio* or birthday* or eid or dussera or "Ganesha Chaturthi" or Holi or "Krishna Janmashtami" or "Maha Shivratri" or Navratri or "Raksha Bandhan" or uposatha or "makha bucha" or "makha puja" or vesak* or "asalha puja" or dhamma or kathina or sanghamitta or "lantern festival" or qingming or "dragon boat" or "double seventh" or "autumn festival" or "double ninth" or "winter solstice" or laba).af. (122754)

42 (birthday* or wedding* or marriage* or "new year*" or hanukkah or chanukah or passover or Shabbat or "rosh chodesh" or "rosh hashanah" or "tzom gedalia" or "yom kippur" or sukkot or "simchat torah" or purim or shavuot).af. (51522)

43 (season* or summer or winter or autumn or winter or school holiday*).af. (415987)

44 ("fiestas decembrinas" or fiesta or navidad or semana santa or pascua).af. (1072)

45 exp Holidays/ (2983)

46 exp Seasons/ (109583)

47 or/41-46 (571436)

48 randomized controlled trial.pt. (524894)

49 controlled clinical trial.pt. (94095)

50 randomi?ed.ab. (612979)

51 placebo.ab. (216013)

52 drug therapy.fs. (2288966)

53 randomly.ab. (352947)

54 trial.ab. (543146)

55 groups.ab. (2165520)

56 (matched communities or matched schools or matched populations).tw. (318)

57 (quasiexperimental or quasi experimental or pseudo experimental).tw. (15235)

58 (nonrandomi?ed or non randomi?ed or pseudo randomi?sed or quasi randomi?ed).tw. (31490)

59 Cluster analysis/ (63845)

60 or/48-59 (5016017)

61 40 and 47 and 60 (21092)

62 exp Weight Loss/ (43162)

63 exp Weight Gain/ (32612)

64 exp Body Weight/ (475199)

65 Overweight/ (26154)

66 Body Mass Index/ (131462)

67 Waist-Hip Ratio/ (4232)

68 Adipose Tissue/ (80908)

69 (weight gain or weight loss).af. (175281)

70 (overweight or over weight or overeat$ or over eat$).af. (89446)

71 weight change$.af. (11931)

72 ((bmi or body mass index or zbmi or weight) adj2 (gain or loss or change or increase or decrease or lower or higher)).af. (232049)

73 or/62-72 (727504)

74 40 and 47 and 60 and 73 (1773)

75 exp animals/ not humans.sh. (4798722)

76 74 not 75 (1378)

***************************

# CENTRAL search strategy:

Database: EBM Reviews - Cochrane Central Register of Controlled Trials <February 2021>

Search Strategy: (589)

--------------------------------------------------------------------------------

1 Dietary Supplements/ (10493)

2 Fasting/ (3192)

3 Overweight/ (5158)

4 Behavior Therapy/ (4615)

5 Social Support/ (3360)

6 Exercise/ (16238)

7 Health Policy/ (202)

8 Health Promotion/ (5952)

9 Health Education/ (3980)

10 ((psychological or behavio?r$) adj (therapy or modif$ or strateg$ or intervention$)).af. (32978)

11 (group therapy or family therapy or cognitive therapy).af. (11926)

12 ((lifestyle or life style) adj (chang$ or intervention$)).af. (7051)

13 counsel?ing.af. (22927)

14 social support.af. (8604)

15 (peer adj2 support).af. (1471)

16 (diet$ adj (modif$ or therapy or intervention$ or strateg$)).af. (9722)

17 (low calorie or calorie control$ or healthy eating).af. (3976)

18 (fasting or modified fast$).af. (35082)

19 (fruit$ or vegetable$).af. (10481)

20 (high fat$ or low fat$ or fatty food$).af. (7069)

21 (aerobics or physical therapy or physical activity or physical inactivity or exercis$).af. (136420)

22 (fitness adj (class$ or regime$ or program$)).af. (302)

23 (physical training or physical education or dance therapy or sendentary behavio?r).af. (6798)

24 (weightwatcher$ or weight watcher$).af. (134)

25 (fat camp$ or diet$ camp$).af. (15)

26 (health promotion or health education).af. (21037)

27 (media intervention$ or community intervention$).af. (845)

28 (family intervention$ or parent$ intervention or health promoting school$).af. (1787)

29 ((school or community) adj2 (program$ or intervention$)).af. (9578)

30 (parent$ adj2 (behavio?r or involve$ or control$ or attitude$ or educat$)).af. (6460)

31 (health polic$ or school polic$ or food polic$ or nutrition polic$).af. (2614)

32 (primary prevention or secondary prevention or preventive measure$ or preventative measure$).af. (12275)

33 (weight gain adj2 prevention).af. (344)

34 (self-weigh* or nutrition or energy restrict$ or program$ or energy reduc$).af. (173968)

35 or/1-34 (369523)

36 (feast* or celebrat* or banquet* or holiday* or vacation* or "special occasion*" or "special event*" or thanksgiving or christmas or easter or ramadan or festiv* or diwali or religio* or birthday* or eid or dussera or "Ganesha Chaturthi" or Holi or "Krishna Janmashtami" or "Maha Shivratri" or Navratri or "Raksha Bandhan" or uposatha or "makha bucha" or "makha puja" or vesak* or "asalha puja" or dhamma or kathina or sanghamitta or "lantern festival" or qingming or "dragon boat" or "double seventh" or "autumn festival" or "double ninth" or "winter solstice" or laba).af. (5826)

37 (birthday* or wedding* or marriage* or "new year*" or hanukkah or chanukah or passover or Shabbat or "rosh chodesh" or "rosh hashanah" or "tzom gedalia" or "yom kippur" or sukkot or "simchat torah" or purim or shavuot).af. (2420)

38 (season* or summer or winter or autumn or winter or school holiday*).af. (15190)

39 ("fiestas decembrinas" or fiesta or navidad or semana santa or pascua).af. (35)

40 exp Holidays/ (23)

41 exp Seasons/ (1028)

42 or/36-41 (22720)

43 exp Weight Loss/ (6445)

44 exp Weight Gain/ (2515)

45 exp Body Weight/ (27922)

46 Overweight/ (5158)

47 Body Mass Index/ (10420)

48 Waist-Hip Ratio/ (269)

49 Adipose Tissue/ (1875)

50 (weight gain or weight loss).af. (32918)

51 (overweight or over weight or overeat$ or over eat$).af. (18679)

52 weight change$.af. (4442)

53 ((bmi or body mass index or zbmi or weight) adj2 (gain or loss or change or increase or decrease or lower or higher)).af. (42248)

54 or/43-53 (68978)

55 35 and 42 and 54 (589)

56 exp animals/ not humans.sh. (12)

57 55 not 56 (589)

***************************

# PsycINFO search strategy:

Database: APA PsycInfo <1806 to March Week 2 2021>

Search Strategy:

--------------------------------------------------------------------------------

1 Cognitive Behavior Therapy/ (20659)

2 exp Cognitive Therapy/ (13587)

3 Dietary Supplements/ (1990)

4 Food deprivation/ (3184)

5 Overweight/ (4542)

6 Obesity/ (25419)

7 Brief psychotherapy/ (5714)

8 Behavior therapy/ (14309)

9 Self-care/ (2747)

10 Social Support/ (37701)

11 Exercise/ (23792)

12 Health Care Policy/ (10891)

13 *prevention/ (21050)

14 Health Promotion/ (25569)

15 Health Education/ (13591)

16 ((psychological or behavio?r$) adj (therapy or modif$ or strateg$ or intervention$)).tw. (63325)

17 (group therapy or family therapy or cognitive therapy).tw. (37696)

18 ((lifestyle or life style) adj (chang$ or intervention$)).tw. (4075)

19 counsel?ing.tw. (90100)

20 social support.tw. (51998)

21 (peer adj2 support).tw. (5550)

22 (diet$ adj (modif$ or therapy or intervention$ or strateg$)).tw. (1498)

23 (low calorie or calorie control$ or healthy eating).tw. (3792)

24 (fasting or modified fast$).tw. (5444)

25 (fruit$ or vegetable$).tw. (20284)

26 (high fat$ or low fat$ or fatty food$).tw. (3571)

27 (aerobics or physical therapy or physical activity or physical inactivity or exercis$).tw. (101995)

28 (fitness adj (class$ or regime$ or program$)).tw. (492)

29 (physical training or physical education or dance therapy or sendentary behavio?r).tw. (7381)

30 (weightwatcher$ or weight watcher$).tw. (60)

31 (fat camp$ or diet$ camp$).tw. (6)

32 (health promotion or health education).tw. (30376)

33 (media intervention$ or community intervention$).tw. (1956)

34 (family intervention$ or parent$ intervention or health promoting school$).tw. (4680)

35 ((school or community) adj2 (program$ or intervention$)).tw. (28910)

36 (parent$ adj2 (behavio?r or involve$ or control$ or attitude$ or educat$)).af. (117916)

37 (health polic$ or school polic$ or food polic$ or nutrition polic$).tw. (11339)

38 (primary prevention or secondary prevention or preventive measure$ or preventative measure$).tw. (9399)

39 (weight gain adj2 prevention).tw. (223)

40 (self-weigh* or nutrition or energy restrict$ or program$ or energy reduc$).tw. (432912)

41 or/1-40 (910066)

42 (feast* or celebrat* or banquet* or holiday* or vacation* or "special occasion*" or "special event*" or thanksgiving or christmas or easter or ramadan or festiv* or diwali or religio* or birthday* or eid or dussera or "Ganesha Chaturthi" or Holi or "Krishna Janmashtami" or "Maha Shivratri" or Navratri or "Raksha Bandhan" or uposatha or "makha bucha" or "makha puja" or vesak* or "asalha puja" or dhamma or kathina or sanghamitta or "lantern festival" or qingming or "dragon boat" or "double seventh" or "autumn festival" or "double ninth" or "winter solstice" or laba).tw. (93691)

43 (birthday* or wedding* or marriage* or "new year*" or hanukkah or chanukah or passover or Shabbat or "rosh chodesh" or "rosh hashanah" or "tzom gedalia" or "yom kippur" or sukkot or "simchat torah" or purim or shavuot).tw. (35906)

44 (season* or summer or winter or autumn or winter or school holiday*).tw. (30169)

45 ("fiestas decembrinas" or fiesta or navidad or semana santa or pascua).tw. (44)

46 exp Holidays/ (453)

47 exp Vacationing/ (610)

48 or/42-47 (153154)

49 Clinical trials/ (11875)

50 Longitudinal studies/ (15873)

51 exp Program evaluation/ (20816)

52 Treatment effectiveness evaluation/ (25519)

53 random$.tw. (211127)

54 (allocat$ or assign$).tw. (131671)

55 ((clinic$ or control$) adj trial$).tw. (77233)

56 ((control$ or experiment$ or intervention$) adj3 group$).tw. (126261)

57 ((singl$ or doubl$ or trebl$ or tripl$) adj3 (blind$ or mask$)).tw. (27149)

58 (crossover$ or "cross over$").tw. (10797)

59 (placebo$ or (usual adj1 treatment$) or wait$ list).tw. (47170)

60 prospectiv$.tw. (71768)

61 (crossover or cross-over).tw. (10712)

62 ((effectiveness or evaluat$) adj3 (stud$ or research$)).tw. (97354)

63 or/49-62 (599735)

64 41 and 48 and 63 (3983)

65 exp Weight Loss/ (3955)

66 exp Weight Gain/ (3235)

67 exp Body Weight/ (55361)

68 Overweight/ (4542)

69 Body Mass Index/ (6259)

70 Adipose Tissue/ (2003)

71 (weight gain or weight loss).tw. (21552)

72 (overweight or over weight or overeat$ or over eat$).tw. (18179)

73 weight change$.tw. (2280)

74 ((bmi or body mass index or zbmi or weight) adj2 (gain or loss or change or increase or decrease or lower or higher)).tw. (27873)

75 or/65-74 (74331)

76 41 and 48 and 63 and 75 (124)

77 exp animals/ not humans.mp. (315633)

78 76 not 77 (121)

***************************

# SciELO search strategy:


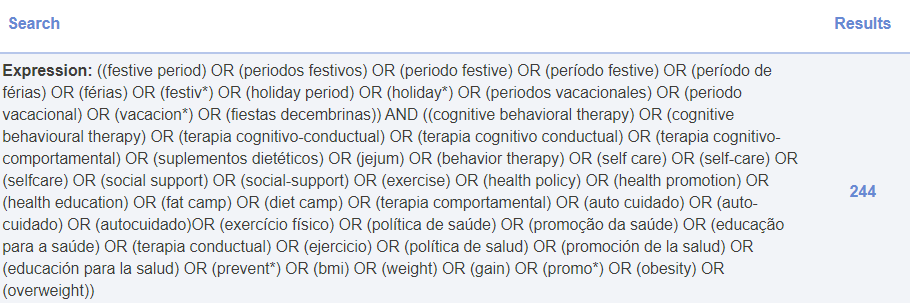


# LILACS search strategy:

("festive period" OR "periodos festivos" OR "periodo festivo" OR "período festivo" OR "período de férias" OR férias OR festiv* OR "holiday period" OR holiday* OR "periodos vacacionales" OR "periodo vacacional" OR vacacion* OR "fiestas decembrinas") AND ("cognitive behavioral therapy" OR "cognitive behavioural therapy" OR "terapia cognitivo-conductual" OR "terapia cognitivo conductual" OR terapia cognitivo-comportamental OR "suplementos dietéticos" OR jejum OR "behavior therapy" OR "self care" OR "self-care" OR selfcare OR "social support" OR "social-support" OR excercise OR "health policy" OR "health promotion" OR "health education" OR "fat camp" OR "diet camp" OR "terapia comportamental" OR "auto cuidado" OR “auto-cuidado” OR autocuidado OR “exercício físico” OR "política de saúde" OR "promoção da saúde" OR "educação para a saúde" OR "terapia conductual" OR ejercicio OR "política de salud" OR "promoción de la salud" OR "educación para la salud" OR prevent* OR bmi OR weight OR gain OR promo* OR obesity OR overweight) AND (db:("LILACS"))

N=222

# Updated MEDLINE Search

Database: Ovid MEDLINE(R) and Epub Ahead of Print, In-Process, In-Data-Review & Other Non-Indexed Citations, Daily and Versions <1946 to January 03, 2023>

Search Strategy:

--------------------------------------------------------------------------------

1 Cognitive Behavioral Therapy/mt [Methods] (14418)

2 Dietary Supplements/ (71864)

3 Fasting/ (38363)

4 Overweight/pc [Prevention & Control] (2220)

5 Obesity/dh, pc [Diet Therapy, Prevention & Control] (24966)

6 exp Psychotherapy, Brief/mt [Methods] (1679)

7 Behavior Therapy/mt [Methods] (11749)

8 Self Care/mt [Methods] (8416)

9 Social Support/ (77711)

10 Exercise/ (137204)

11 Health Policy/ (71656)

12 Primary Prevention/mt [Methods] (6991)

13 Health Promotion/ (80348)

14 Health Education/ (63282)

15 ((psychological or behavio?r$) adj (therapy or modif$ or strateg$ or intervention$)).af. (97334)

16 (group therapy or family therapy or cognitive therapy).af. (19952)

17 ((lifestyle or life style) adj (chang$ or intervention$)).af. (20026)

18 counsel?ing.af. (161298)

19 social support.af. (103647)

20 (peer adj2 support).af. (7317)

21 (diet$ adj (modif$ or therapy or intervention$ or strateg$)).af. (36859)

22 (low calorie or calorie control$ or healthy eating).af. (13808)

23 (fasting or modified fast$).af. (139266)

24 (fruit$ or vegetable$).af. (208918)

25 (high fat$ or low fat$ or fatty food$).af. (66819)

26 (aerobics or physical therapy or physical activity or physical inactivity or exercis$).af. (659504)

27 (fitness adj (class$ or regime$ or program$)).af. (1055)

28 (physical training or physical education or dance therapy or sendentary behavio?r).af. (61364)

29 (weightwatcher$ or weight watcher$).af. (264)

30 (fat camp$ or diet$ camp$).af. (30)

31 (health promotion or health education).af. (244808)

32 (media intervention$ or community intervention$).af. (3072)

33 (family intervention$ or parent$ intervention or health promoting school$).af. (3235)

34 ((school or community) adj2 (program$ or intervention$)).af. (46824)

35 (parent$ adj2 (behavio?r or involve$ or control$ or attitude$ or educat$)).af. (44497)

36 (health polic$ or school polic$ or food polic$ or nutrition polic$).af. (189497)

37 (primary prevention or secondary prevention or preventive measure$ or preventative measure$).af. (105108)

38 (weight gain adj2 prevention).af. (620)

39 (self-weigh* or nutrition or energy restrict$ or program$ or energy reduc$).af. (2340486)

40 or/1-39 (3840935)

41 (feast* or celebrat* or banquet* or holiday* or vacation* or "special occasion*" or "special event*" or thanksgiving or christmas or easter or ramadan or festiv* or diwali or religio* or birthday* or eid or dussera or "Ganesha Chaturthi" or Holi or "Krishna Janmashtami" or "Maha Shivratri" or Navratri or "Raksha Bandhan" or uposatha or "makha bucha" or "makha puja" or vesak* or "asalha puja" or dhamma or kathina or sanghamitta or "lantern festival" or qingming or "dragon boat" or "double seventh" or "autumn festival" or "double ninth" or "winter solstice" or laba).af. (136434)

42 (birthday* or wedding* or marriage* or "new year*" or hanukkah or chanukah or passover or Shabbat or "rosh chodesh" or "rosh hashanah" or "tzom gedalia" or "yom kippur" or sukkot or "simchat torah" or purim or shavuot).af. (54806)

43 (season* or summer or winter or autumn or winter or school holiday*).af. (455197)

44 ("fiestas decembrinas" or fiesta or navidad or semana santa or pascua).af. (1210)

45 exp Holidays/ (3232)

46 exp Seasons/ (118395)

47 or/41-46 (626559)

48 randomized controlled trial.pt. (582899)

49 controlled clinical trial.pt. (95140)

50 randomi?ed.ab. (700538)

51 placebo.ab. (234738)

52 drug therapy.fs. (2562157)

53 randomly.ab. (397673)

54 trial.ab. (629177)

55 groups.ab. (2450054)

56 (matched communities or matched schools or matched populations).tw. (359)

57 (quasiexperimental or quasi experimental or pseudo experimental).tw. (18903)

58 (nonrandomi?ed or non randomi?ed or pseudo randomi?sed or quasi randomi?ed).tw. (36563)

59 Cluster analysis/ (68536)

60 or/48-59 (5631041)

61 40 and 47 and 60 (24078)

62 exp Weight Loss/ (48507)

63 exp Weight Gain/ (35737)

64 exp Body Weight/ (519615)

65 Overweight/ (31421)

66 Body Mass Index/ (147095)

67 Waist-Hip Ratio/ (4550)

68 Adipose Tissue/ (87670)

69 (weight gain or weight loss).af. (196108)

70 (overweight or over weight or overeat$ or over eat$).af. (102691)

71 weight change$.af. (13508)

72 ((bmi or body mass index or zbmi or weight) adj2 (gain or loss or change or increase or decrease or lower or higher)).af. (261339)

73 or/62-72 (801585)

74 40 and 47 and 60 and 73 (1950)

75 exp animals/ not humans.sh. (5086989)

76 74 not 75 (1523)

77 limit 76 to yr="2021 -Current" (147)

***************************

# Table S1. Table of search results by database and supplementary search

| **Database** | **Date searched and exported** | **No. refs found** | **Date duplicates removed** | **No. refs after duplicates removed** | **Potentially meets the inclusion criteria** | **Include** | **Primary** | **Secondary** | **Ongoing** |
| --- | --- | --- | --- | --- | --- | --- | --- | --- | --- |
| ***Electronic Databases*** |  |  |  |  |  |  |  |  |  |
| EMBASE | 15/03/21 | 1505 | 21/03/21 | 1194 | 44 | **4** | 1 | 1 | 2 |
| Medline (Ovid) | 10/03/21 | 1378 | 21/03/21 | 1376 | 61 | **12** | 10 | 2 | 0 |
| Medline (Ovid) Updated Search | 04/01/23 | 147 | 04/01/23 | 102 | 1 | **0** | 0 | 0 | 0 |
| LILACS (BVSalud) | 10/03/21 | 222 | 21/03/21 | 187 | 4 | **0** | 0 | 0 | 0 |
| SciELO | 10/03/21 | 244 | 21/03/21 | 226 | 3 | **0** | 0 | 0 | 0 |
| Cochrane CENTRAL | 11/03/21 | 589 | 21/03/21 | 245 | 28 | **9** | 0 | 6 | 3 |
| PsycINFO | 13/03/21 | 121 | 21/03/21 | 75 | 9 | **1** | 0 | 1 | 0 |
| **Subtotal:** |  | **4206** |  | **3405** | **150** | **26** | **11** | **10** | **5** |
| ***Supplementary searches*** |  |  |  |  |  |  |  |  |  |
| Google and Google Scholar; ClinicalTrials.gov; reference list of included studies; reference list of systematic reviews, experts. | 30/11/22 |  |  | **10** | **10** | **5** | **1** | **0** | **4** |
| **TOTAL:** |  | **4206** |  | **3415** | **160** | **31** | **12** | **10** | **9** |

# File S2. List of ongoing studies (n = 9)

| **Number** | **Reference** |
| --- | --- |
| 1 | Goldstein T, Serok E, Kark JD. Joint parent-children nutritional activities may improve BMI in children who are overweight or obese. Annals of Nutrition and Metabolism. 2015;1):427. |
| 2 | Moreno JP, Dadabhoy H, Musaad S, Baranowski T, Thompson D, Alfano CA, et al. Evaluation of a Circadian Rhythm and Sleep-Focused Mobile Health Intervention for the Prevention of Accelerated Summer Weight Gain Among Elementary School–Age Children: Protocol for a Randomized Controlled Feasibility Study. JMIR Res Protoc. 2022;11(5):e37002. |
| 3 | Morton G, Schieder C, Kaiser L. Anthropometric outcomes related to selfsatisfaction, parent and peer support in overweight youth participating in a fitness and nutrition themed summer camp. FASEB Journal Conference: Experimental Biology. 2015;29(1 Meeting Abstracts). |
| 4 | ClinicalTrials.gov. NCT00974727, A Gardening Program to Assess Unhealthy Lifestyle Contributions to Summer Weight Gain in Children. https://clinicaltrialsgov/show/NCT00974727. 2009. |
| 5 | ClinicalTrials.gov. NCT04072549, Reducing Health Disparities in Childhood Obesity. https://clinicaltrialsgov/show/NCT04072549. 2019. |
| 6 | ClinicalTrials.gov. NCT04544137, Summer Food Service Program. https://clinicaltrialsgov/show/NCT04544137. 2020. |
| 7 | ClinicalTrials.gov. NCT05060978, Evaluation of the Effect of Two Online Interventions -Watch Your Weight During the Holidays Program and the Relative 5:2 Fasting- for the Prevention of Body Weight Gain at 8 Weeks in Mexican Adults in the Covid-19 Pandemic. https://clinicaltrialsgov/ct2/show/NCT05060978. 2021. |
| 8 | ClinicalTrials.gov. NCT05580926, Evaluation of the Efficacy of the Watch Your Weight During Holidays Program for the Prevention of Body Weight Gain in Mexican Adults. https://clinicaltrialsgov/ct2/show/NCT05580926. 2022. |
| 9 | ClinicalTrials.gov. NCT05367674, Summer Harvest Adventure: A Garden-based Obesity Prevention Program for Children Residing in Low-resource Communities (SHA). https://clinicaltrialsgov/ct2/show/NCT05367674. 2022. |

# File S3. List of excluded studies (n=129), with reason for exclusion

| **No.** | **Reference** | **Reason – Detail** |
| --- | --- | --- |
| 1 | Abreu CM, Williams E, Smith AJ, Pak E, Medina E, Malika NM, et al. Exercise or diet? which is a better predictor of body mass index in children? Journal of Investigative Medicine. 2018;66 (1):65. | Study type- Doesn´t mention a control group. |
| 2 | Aksungar FB, Topkaya AE, Akyildiz M. Interleukin-6, C-reactive protein and biochemical parameters during prolonged intermittent fasting. Annals of Nutrition and Metabolism. 2007;51(1):88-95. | Intervention- Not aimed to prevent weight gain during festive or holiday period |
| 3 | Alayafi YR. The physiological effect of intermittent fasting (fasting the month of ramadan) on anthropometrics and blood variables. Dissertation Abstracts International: Section B: The Sciences and Engineering. 2015;75(12-B(E)):No Pagination Specified. | Study type- Not a RCT or nRCT (there is no control group) |
| 4 | Alharbi T, Yue DK, Wong J, Markovic T, Wu T, Brooks BA, et al. Ramadan as a model of intermittent fasting: Effects on gut hormones, appetite, and body composition in subjects with and without type 2 diabetes mellitus. Diabetes. 2014;(1):A486-A7. | Participants- One group with only T2DM participants Study type- Doesn't seem to be an RCT |
| 5 | Alsubheen SA, Ismail M, Baker A, Blair J, Adebayo A, Kelly L, et al. The effects of diurnal Ramadan fasting on energy expenditure and substrate oxidation in healthy men. Br J Nutr. 2017;118(12):1023-30. | Intervention- Not aimed to prevent weight gain |
| 6 | Attarzadeh Hosseini SR, Sardar MA, Hejazi K, Farahati S. The effect of ramadan fasting and physical activity on body composition, serum osmolarity levels and some parameters of electrolytes in females. Int J Endocrinol Metab. 2013;11(2):88-94. | Intervention- Not aimed to prevent weight gain |
| 7 | Banzer W, Berg A, Braumann K, Fuhrer-Sakel D, Halle M, Martin S, et al. Benefits of the ACOORH concept on weight control and metabolic regulation after 12 weeks of intervention: Results of a multicenter RCT. Obes Facts. 2018;11 (Supplement 1):313. | Intervention- Not an intervention during holidays or festive periods |
| 8 | Bassami M, Ahmadizad S, Tahmasebi W, Khedmatgozar E, Rokhsati S. Effects of Ramadan fasting and regular exercise training on fat and CHO metabolism. Iranian Journal of Endocrinology and Metabolism. 2013;15(4):360-9. | Intervention- Not aimed to prevent weight gain during festive or holiday periods |
| 9 | Ben Jemaa H, Mahjoub F, Berriche O, Gammoudi A, Chaabouni S, Jamoussi H. Impacts of nutritional education before Ramadan fasting on dietary intake, weight and body composition in diabetic patients. Tunis Med. 2019;97(10):1139-45. | Participants- Participants with diabetes |
| 10 | Bencharif M, Boudaoud C, Fenaghra A, Benabbas Y. Effect of pre-ramadan education on dietary intake and anthropometry-comparison between two groups of diabetic patients. Romanian Journal of Diabetes, Nutrition and Metabolic Diseases. 2017;24(4):295-307. | Participants- Participants with diabetes |
| 11 | Benestad B, Karlsen TI, Smastuen MC, Lekhal S, Hertel JK, Steinsbekk S, et al. Health-related quality of life after camp-based family obesity treatment: An RCT. BMJ Paediatrics Open. 2019;3 (1) (no pagination)(e000413). | Intervention- Aimed to treat obesity (weight loss) |
| 12 | Benestad B, Lekhal S, Hertel JK, Odegard R, Hjelmesaeth J. Long-term effectiveness of two family based life style intervention programs on childhood obesity. A 2-years randomized controlled pragmatic trial. Obes Facts. 2014;7(131). | Other- Conference abstract not all data available to decide. Can't decide to include, not enough information. |
| 13 | Benestad B, Lekhal S, Smastuen MC, Hertel JK, Halsteinli V, Odegard RA, et al. Camp-based family treatment of childhood obesity: randomised controlled trial. Arch Dis Child. 2017;102(4):303-10. | Intervention- Not aimed to prevent weight gain |
| 14 | Berkowitz SA, O'Neill J, Sayer E, Shahid NN, Petrie M, Schouboe S, et al. A community supported agriculture intervention for health center patients: A randomized clinical trial. Journal of General Internal Medicine. 2019;34 (2 Supplement):S107-S8. | Intervention- Not aimed to prevent weight gain |
| 15 | Bernstein R, Schneider R, Welch W, Dressel A, DeNomie M, Kusch J, et al. Biking for Health: Results of a Pilot Randomized Controlled Trial Examining the Impact of a Bicycling Intervention on Lower-Income Adults. Wmj. 2017;116(3):154-60. | Intervention- Not aimed to prevent weight gain |
| 16 | Bertz F, Pacanowski C, Levitsky D. Self-weighing to prevent age-related weight gain in young adults. FASEB Journal Conference: Experimental Biology. 2014;28(1 SUPPL. 1). | Intervention - Not aimed to prevent weight gain during festive or holiday period |
| 17 | Bezerra P, Rodrigues LP, Ayan C, Cancela JM. The influence of winter and summer seasons on physical fitness in aged population. Archives of Gerontology and Geriatrics. 2018;76:80-4. | Study type- Not a RCT, nRCT or Cluster RCT (there is no control group) |
| 18 | Black DR. Weight changes in a couples program: negative association of marital adjustment. J Behav Ther Exp Psychiatry. 1988;19(2):103-11. | Intervention- Weight loss intervention |
| 19 | Boardley D, Fahlman M, Topp R, Morgan AL, McNevin N. The impact of exercise training on blood lipids in older adults. Am J Geriatr Cardiol. 2007;16(1):30-5. | Intervention- Not aimed to prevent weight gain during festive or holiday period |
| 20 | Boutelle KN, Kirschenbaum DS, Baker RC, Mitchell ME. How can obese weight controllers minimize weight gain during the high risk holiday season? By self-monitoring very consistently. Health Psychol. 1999;18(4):364-8. | Intervention- Can't decide to include, it seems that participants were taking a weight loss program at the same time with the study of prevention. |
| 21 | Braet C, Van Winckel M. Long-term follow-up of a cognitive behavioral treatment program for obese children. Behavior Therapy. 2000;31(1):55-74. | Intervention- Weight loss intervention |
| 22 | Braet C, Van Winckel M, Van Leeuwen K. Follow-up results of different treatment programs for obese children. Acta Paediatr. 1997;86(4):397-402. | Intervention- Seems to be a weight loss intervention |
| 23 | Brehm BJ, Lattin BL, Summer SS, Boback JA, Gilchrist GM, Jandacek RJ, et al. One-year comparison of a high-monounsaturated fat diet with a high-carbohydrate diet in type 2 diabetes. Diabetes Care. 2009;32(2):215-20. | Participants- Participants with diabetes Intervention- Not aimed to prevent weight gain |
| 24 | Bunout D, Barrera G, De La Maza P, Gattas V, Hirsch S. Seasonal variation in insulin sensitivity in healthy elderly people. Nutrition. 2003;19(4):310-6. | Intervention- Not aimed to prevent weight gain |
| 25 | Burke V, Giangiulio N, Gillam HF, Beilin LJ, Houghton S. Physical activity and nutrition programs for couples: A randomized controlled trial. J Clin Epidemiol. 2003;56(5):421-32. | Intervention- Not during holiday or festive period |
| 26 | Carrel AL, Clark RR, Peterson S, Eickhoff J, Allen DB. School-based fitness changes are lost during the summer vacation. Arch Pediatr Adolesc Med. 2007;161(6):561-4. | Study type- Not a RCT, nRCT or Cluster RCT (there is no control group) |
| 27 | Carson RL, Castelli DM, Pulling Kuhn AC, Moore JB, Beets MW, Beighle A, et al. Impact of trained champions of comprehensive school physical activity programs on school physical activity offerings, youth physical activity and sedentary behaviors. Prev Med. 2014;69(S):S12-S9. | Intervention- Not aimed to prevent weight gain |
| 28 | Chen HS, Wu TE, Jap TS, Chen RL, Lin HD. Effects of health education on glycemic control during holiday time in patients with type 2 diabetes mellitus. Am J Manag Care. 2008;14(1):45-51. | Participants- Participants with diabetes Intervention- Not aimed to prevent weight gain |
| 29 | Cooper JA, Tokar T. A prospective study on vacation weight gain in adults. Physiol Behav. 2016;156:43-7. | Study type- Not a RCT, nRCT or cluster RCT; Cohort study |
| 30 | Cristi-Montero C. ¿Como prevenir el aumento de peso durante las vacaciones de fiestas patrias en escolares chilenos?. Rev chil nutr. 2011;38(4):501-2. | Study type- Not a RCT, nRCT or cluster RCT; Letter to editor |
| 31 | Cristi-Montero C, Bresciani G, Alvarez A, Arriagada V, Beneventi A, Canepa V, et al. Periodos críticos en la variación de la composición corporal en niños en edad escolar. Nutrición Hospitalaria. 2014;30(4):782-6. | Intervention- There was no intervention |
| 32 | Culha C, Gorar S, Aral Y. Circulating obestatin levels during ramadan fasting in normal weight and obese subjects. Kuwait Medical Journal. 2019;51(4):335-40. | Intervention- There was no intervention Study type- Not a RCT, nRCT or cluster RCT |
| 33 | De Bock F, Genser B, Raat H, Fischer JE, Renz-Polster H. A participatory physical activity intervention in preschools: A cluster randomized controlled trial. Am J Prev Med. 2013;45(1):64-74. | Participants- ages below 6 years Intervention- Not aimed to prevent weight gain during holiday or festive periods |
| 34 | Deforche B, De Bourdeaudhuij I, Tanghe A, Debode P, Hills AP, Bouckaert J. Post-treatment phone contact: a weight maintenance strategy in obese youngsters. Int J Obes. 2005;29(5):543-6. | Intervention- Not aimed to prevent weight gain during holiday or festive periods |
| 35 | Dewanti L, Watanabe C, Sulistiawati, Ohtsuka R. Unexpected changes in blood pressure and hematological parameters among fasting and nonfasting workers during Ramadan in Indonesia. Eur J Clin Nutr. 2006;60(7):877-81. | Intervention- There was no intervention; it seems to be an exposure Study design- Not a RCT, nRCT or cluster RCT; it seems to be an observational study |
| 36 | DiGiacinto K. Pe followed me home from school today: Effects of the activity challenge on youth summer physical activity. Dissertation Abstracts International Section A: Humanities and Social Sciences. 2011;71(9-A):3203. | Intervention- Not aimed to prevent weight gain during holiday or festive period Study design- Not a RCT or nRCT (ther is no control group) |
| 37 | Dobos G, Cramer H, Lauche R, Klose P, Saddat C, Fathi I, et al. Effects of a modified ramadan fasting on physical and mental health in healthy adult muslims-a randomized controlled trial. Global Advances in Health and Medicine. 2018;7:259-60. | Intervention- Not aimed to prevent weight gain during holiday or festive period |
| 38 | Domenghini CM. Physical activity and curriculum development of an after-school gardening program for youth health. Dissertation Abstracts International: Section B: The Sciences and Engineering. 2012;73(5-B):2588. | Study type- Not a RCT or nRCT (ther is no control group) |
| 39 | Dzator JA, Hendrie D, Burke V, Gianguilio N, Gillam HF, Beilin LJ, et al. A randomized trial of interactive group sessions achieved greater improvements in nutrition and physical activity at a tiny increase in cost. J Clin Epidemiol. 2004;57(6):610-9. | Intervention- Not aimed to prevent weight gain during holiday or festive period |
| 40 | Fahey MC, Klesges RC, Kocak M, Talcott GW, Krukowski RA. Seasonal fluctuations in weight and self-weighing behavior among adults in a behavioral weight loss intervention. Eating and weight disorders. 2019. | Intervention - Study with a weight loss goal |
| 41 | Faude O, Kerper O, Multhaupt M, Winter C, Beziel K, Junge A, et al. Football to tackle overweight in children. Scand J Med Sci Sports. 2010;20 Suppl 1:103-10. | Intervention- Not aimed to prevent weight gain during holiday or festive period |
| 42 | Fitzgibbon ML, Stolley MR, Schiffer L, Van Horn L, KauferChristoffel K, Dyer A. Hip-Hop to Health Jr. for Latino preschool children. Obesity (Silver Spring). 2006;14(9):1616-25. | Intervention- Not aimed to prevent weight gain during holiday or festive period |
| 43 | Gately PJ, Cooke CB, Barth JH, Bewick BM, Radley D, Hill AJ. Children's residential weight-loss programs can work: a prospective cohort study of short-term outcomes for overweight and obese children. Pediatrics. 2005;116(1):73-7. | Intervention- Not aimed to prevent weight gain during holiday or festive period |
| 44 | George GL, Schneider C, Martin A, Ginsburg D, Kaiser L. Individualized feedback during summer camp influences anthropometric changes in california's central valley overweight youth. FASEB Journal Conference: Experimental Biology. 2013;27(Meeting Abstracts). | Intervention- Not aimed to prevent weight gain during holiday or festive period |
| 45 | Ghashang SK, Raha S, Hamdan I, Gutenbrunner C, Nugraha B. A prospective controlled study on Ramadan fasting in the healthy young males in summer in Germany: Effect on cytokines. Journal of Complementary and Integrative Medicine. 2020;(no pagination)(20190209). | Other- Conference abstract not all data available to decide. Can't decide to include, not enough information." |
| 46 | Ghatrehsamani S, Khavarian N, Beizaei M, Ramedan R, Poursafa P, Kelishadi R. Effect of different physical activity training methods on overweight adolescents. ARYA Atheroscler. 2010;6(2):45-9. | Intervention- Not aimed to prevent weight gain during holiday or festive period |
| 47 | Gorely T, Nevill ME, Morris JG, Stensel DJ, Nevill A. Effect of a school-based intervention to promote healthy lifestyles in 7-11 year old children. The International Journal of Behavioral Nutrition and Physical Activity Vol 6 2009, ArtID 5. 2009;6. | Intervention- Not aimed to prevent weight gain during holiday or festive period |
| 48 | Group HS, Mobley CC, Stadler DD, Staten MA, El Ghormli L, Gillis B, et al. Effect of nutrition changes on foods selected by students in a middle school-based diabetes prevention intervention program: the HEALTHY experience. J Sch Health. 2012;82(2):82-90. | Intervention- Not aimed to prevent weight gain during holiday or festive period |
| 49 | Haghdoost AA, PoorRanjbar M. The interaction between physical activity and fasting on the serum lipid profile during Ramadan. Singapore Med J. 2009;50(9):897-901. | Intervention- Not aimed to prevent weight gain during holiday or festive period |
| 50 | Heelan KA, Abbey BM, Donnelly JE, Mayo MS, Welk GJ. Evaluation of a walking school bus for promoting physical activity in youth. J Phys Act Health. 2009;6(5):560-7. | Intervention- Not during holiday or festive period |
| 51 | Helander EE, Wansink B, Chieh A. Weight Gain over the Holidays in Three Countries. N Engl J Med. 2016;375(12):1200-2. | Study type- Not a RCT, nRCT or Clúster RCT |
| 52 | Herget S, Reichardt S, Grimm A, Petroff D, Kapplinger J, Haase M, et al. High-Intensity Interval Training for Overweight Adolescents: Program Acceptance of a Media Supported Intervention and Changes in Body Composition. Int J Environ Res Public Health. 2016;13(11):08. | Intervention- Not aimed to prevent weight gain during holiday or festive period |
| 53 | Ickovics JR, Duffany KO, Shebl FM, Peters SM, Read MA, Gilstad-Hayden KR, et al. Implementing School-Based Policies to Prevent Obesity: Cluster Randomized Trial. Am J Prev Med. 2019;56(1):e1-e11. | Intervention- Not aimed to prevent weight gain during holiday or festive period |
| 54 | ISRCTN96347873, STockholm Obesity Prevention Project. http://wwwwhoint/trialsearch/Trial2aspx?TrialID=ISRCTN96347873. 2008. | Intervention- Not aimed to prevent weight gain during holiday or festive period |
| 55 | ISRCTN14385822, Magic breakfast: evaluating school breakfast provision. http://wwwwhoint/trialsearch/Trial2aspx?TrialID=ISRCTN14385822. 2016. | Intervention- Not aimed to prevent weight gain during holiday or festive period |
| 56 | ISRCTN11371954, VisezEauA(R) (ReachforWater): a project to learn how to increase drinking of tap water and reduce drinking of bottled and sugary drinks among primary school children, with the aim of encouraging healthy weight and protecting the environment. http://wwwwhoint/trialsearch/Trial2aspx?TrialID=ISRCTN11371954. 2020. | Intervention- Not aimed to prevent weight gain during festive or holiday period |
| 57 | Johnston CA, El-Mubasher A, Palcic JL, Woehler D, Foreyt J. Impact of ethnicity on weight based outcomes for elementary school aged children. Obesity (Silver Spring). 2010;2):S200. | Other- Conference abstract not all data available to decide. Can't decide to include, not enough information. |
| 58 | Kain J, Leyton B, Cerda R, Vio F, Uauy R. Two-year controlled effectiveness trial of a school-based intervention to prevent obesity in Chilean children. Public Health Nutr. 2009;12(9):1451-61. | Intervention- Not aimed to prevent weight gain during festive or holiday period |
| 59 | Karatoprak C, Yolbas S, Cakirca M, Cinar A, Zorlu M, Kiskac M, et al. The effects of long term fasting in Ramadan on glucose regulation in type 2 diabetes mellitus. Eur Rev Med Pharmacol Sci. 2013;17(18):2512-6. | Participants- Participants with diabetes Intervention- Not aimed to prevent weight gain |
| 60 | Kellam S, Olvera N, Leung P, Liu J, Smith D. Effect of exercise dosages on adiposity indices in overweight girls. Salud Publica Mex. 2013;55 Suppl 3:415-21. | Intervention- Aimed to reduce adiposity rather than prevent weight gain during holiday or festive period |
| 61 | Kellam S, Olvera N, Liu J, Smith D, Leung P. Physical activity dosage required to reduce adiposity indicators in overweight minority girls. Obesity (Silver Spring). 2010;2):S135. | Intervention- Not aimed to prevent weight gain during holiday or festive period |
| 62 | Kilanowski JF. Nutrition & physical education in the summer migrant classroom (MEP). Clinical and Translational Science. 2010;3 (2):S30-S1. | Intervention- It doesn't seems to be aimed to prevent weight gain. Other than that I think that It could be potential. |
| 63 | Kitzman H, Dodgen L, Mamun A, Slater JL, King G, Slater D, et al. Community-based participatory research to design a faith-enhanced diabetes prevention program: The Better Me Within randomized trial. Contemp Clin Trials. 2017;62:77-90. | Intervention- Not aimed to prevent weight gain during festive or holiday period Intervention- Intervention with a weight loss aim |
| 64 | Knowlden AP. Feasibility and efficacy of the Enabling Mothers to Prevent Pediatric Obesity Through Web-Based Education and Reciprocal Determinism (EMPOWER) randomized control trial. Dissertation Abstracts International Section A: Humanities and Social Sciences. 2014;75(2-A(E)):No Pagination Specified. | Intervention- Not aimed to prevent weight gain during festive or holiday period |
| 65 | Kramer EN. "S.P.L.A.S.H. into fitness!" an identity-focused behavioral swim camp and family-oriented ehealth intervention for girls. Dissertation Abstracts International: Section B: The Sciences and Engineering. 2020;81(10-B):No Pagination Specified. | Intervention- Not aimed to prevent weight gain during festive or holiday period |
| 66 | Kuroko S, Black K, Chryssidis T, Finigan R, Hann C, Haszard J, et al. Create our own kai: A randomised control trial of a cooking intervention with group interview insights into adolescent cooking behaviours. Nutrients. 2020;12 (3) (no pagination)(796). | Intervention- Not aimed to prevent weight gain during festive or holiday period |
| 67 | Laghaei N, Nikseresht M, Taheri-Kalani A. Interleukin-7 and aerobic capacity responses after 20 sessions of low-intensity resistance training in fasting state during ramadan. [Persian]. Journal of Isfahan Medical School. 2020;38(579):393-400. | Intervention- Not aimed to prevent weight gain during festive or holiday period |
| 68 | Lauche R, Fathi I, Saddat C, Klose P, Al-Abtah J, Bussing A, et al. The effects of Ramadan fasting on physical and mental health in healthy adult Muslims-Study protocol for a randomised controlled trial. Advances in Integrative Medicine. 2016;3(1):26-30. | Intervention- Not aimed to prevent weight gain during festive or holiday period |
| 69 | Lloyd JJ, Wyatt KM, Creanor S. Behavioural and weight status outcomes from an exploratory trial of the Healthy Lifestyles Programme (HeLP): a novel school-based obesity prevention programme. BMJ Open. 2012;2(3). | Intervention- Not aimed to prevent weight gain during festive or holiday period |
| 70 | López R, Cisternas-Vallejos P, Devaud Y, Muñoz-Cofré R, Gómez-Bruton A, Lizana PA. Can Elastic Band Resistance Training Programs Mitigate Holiday Weight Gain and Improve Hand-Grip Strength in Older Women? Int j morphol. 2020;38(5):1173-8. | Study type- Not a RCT, nRCT or Clúster RCT |
| 71 | Maislos M, Abou-Rabiah Y, Zuili I, Iordash S, Shany S. Gorging and plasma HDL-cholesterol - The Ramadan model. Eur J Clin Nutr. 1998;52(2):127-30. | Intervention- Not aimed to prevent weight gain during festive or holiday period. |
| 72 | Matsuhashi T, Sano M, Fukuda K, Kohsaka S, Suzuki Y. Sitagliptin counteracts seasonal fluctuation of glycemic control. World Journal of Diabetes. 2012;3(6):118-22. | Participants- Study with type 2 diabetes participants Intervention- Not aimed to prevent weight gain during festive or holiday period |
| 73 | Matvienko O, Ahrabi-Fard I. The effects of a 4-week after-school program on motor skills and fitness of kindergarten and first-grade students. Am J Health Promot. 2010;24(5):299-303. | Participants- Children in ages below 6 years Intervention- Not aimed to prevent weight gain during festive or holiday period |
| 74 | Maughan RJ, Bartagi Z, Dvorak J, Zerguini Y. Dietary intake and body composition of football players during the holy month of Ramadan. J Sports Sci. 2008;26(SUPPL. 3):S29-S38. | Intervention- Not aimed to prevent weight gain during festive or holiday period |
| 75 | McMinn D, Rowe DA, Murtagh S, Nelson NM. The effect of a school-based active commuting intervention on children's commuting physical activity and daily physical activity. Prev Med. 2012;54(5):316-8. | Intervention- Intervention lasted less than two weeks. Intervention- Not aimed to prevent weight gain during festive or holiday period |
| 76 | Meucci M, Baldari C, Guidetti L, Alley JR, Cook C, Collier SR. Metabolomic Shifts Following Play-Based Activity in Overweight Preadolescents. Current Pediatric Reviews. 2017;13(2):144-51. | Intervention- Not aimed to prevent weight gain during festive or holiday period |
| 77 | Meucci M, Curry CD, Baldari C, Guidetti L, Cook C, Collier SR. Effect of play-based summer break exercise on cardiovascular function in adolescents. Acta Paediatrica, International Journal of Paediatrics. 2013;102(1):e24-e8. | Intervention- Not aimed to prevent weight gain during festive or holiday period |
| 78 | Moazami M, Bijeh N, Abbasian S. A comparison of the effects of ramadan fasting and regular aerobic exercise on 70-Kda heat shock protein (Hsp70), lipid profiles and resistance insulin in non-active obese men. [Persian]. Iranian Journal of Endocrinology and Metabolism. 2013;15(1):67-77. | Intervention- Not aimed to prevent weight gain during festive or holiday period |
| 79 | Nagy LC, Horne M, Faisal M, Mohammed MA, Barber SE. Ethnic differences in sedentary behaviour in 6-8-year-old children during school terms and school holidays: a mixed methods study. BMC Public Health. 2019;19(1):152. | Intervention- Study focused on sedentary beahiors, rather than weight gain prevention |
| 80 | ClinicalTrials.gov. NCT00306449, Prevention of Weight Gain in Young Adults. https://clinicaltrialsgov/show/NCT00306449. 2006. | Intervention- Not aimed to prevent weight gain during festive or holiady period |
| 81 | ClinicalTrials.gov. NCT00365651, Nutrition and Soccer for Pediatric Overweight and Obesity. https://clinicaltrialsgov/show/NCT00365651. 2006. | Intervention- Aimed to reduce BMI |
| 82 | ClinicalTrials.gov. NCT00893529, A Study of the Effect of Replacing Sugary Drinks by Low-sugar Alternatives on Body Weight and Fat Mass in Children. https://clinicaltrialsgov/show/NCT00893529. 2009. | Intervention- Not aimed to prevent weight gain during festive or holiday period |
| 83 | ClinicalTrials.gov. NCT01039116, Taking Action Together- A Diabetes Prevention Program. https://clinicaltrialsgov/show/NCT01039116. 2009. | Intervention- Not aimed to prevent weight gain during festive or holiday period |
| 84 | ClinicalTrials.gov. NCT01373762, The Fitness, Game Bike Adherence, Motivation and Exercise Study. https://clinicaltrialsgov/show/NCT01373762. 2011. | Intervention- Not aimed to prevent weight gain during festive or holiday period Ohter- Not enought information |
| 85 | ClinicalTrials.gov. NCT01290016, Youth Lifestyle Intervention With Food and Exercise. https://clinicaltrialsgov/show/NCT01290016. 2011. | Intervention- Not aimed to prevent weight gain during festive or holiday period |
| 86 | ClinicalTrials.gov. NCT02187939, Get in the GROOVE! https://clinicaltrialsgov/show/NCT02187939. 2014. | Participants- No restriction in the medical conditions that participants could have (e.g. Diabetes, CKD, etc.) |
| 87 | ClinicalTrials.gov. NCT02908230, Camp NERF: methods of a Summer Nutrition Ed Rec & Fitness Program to Prevent Unhealthy Weight Gain in Children. https://clinicaltrialsgov/show/NCT02908230. 2016. | Participants- This study includes children of 4 y to 12 y. |
| 88 | ClinicalTrials.gov. NCT02770196, Intervention Study of Cost-Offset Community Supported Agriculture (CO-CSA). https://clinicaltrialsgov/show/NCT02770196. 2016. | Intervention- Not aimed to prevent weight gain during festive or holiday period |
| 89 | ClinicalTrials.gov. NCT03805295, Implementation and Evaluation of a Before School Physical Activity Program in Revere, MA. https://clinicaltrialsgov/show/NCT03805295. 2019. | Intervention- Not aimed to prevent weight gain during festive or holiday period |
| 90 | ClinicalTrials.gov. NCT04095910, Evaluation of the Planet Nutrition Program on Obesity Parameters in Mexican Schoolchildren. https://clinicaltrialsgov/show/NCT04095910. 2019. | Intervention- Not aimed to prevent weight gain during festive or holiday period |
| 91 | ClinicalTrials.gov. NCT04608188, Preventing Weight Gain and Unhealthy Behaviors in Children. https://clinicaltrialsgov/show/NCT04608188. 2020. | Participants- Ages below 6 years |
| 92 | Nugraha B, Ghashang SK, Hamdan I, Gutenbrunner C. Effect of Ramadan fasting on fatigue, mood, sleepiness, and health-related quality of life of healthy young men in summer time in Germany: A prospective controlled study. Appetite. 2017;111:38-45. | Intervention- The study seems to not have an intervention, it is more like an expossure |
| 93 | Peck KY, DiStefano LJ, Marshall SW, Padua DA, Beutler AI, de la Motte SJ, et al. Effect of a Lower Extremity Preventive Training Program on Physical Performance Scores in Military Recruits. J Strength Cond Res. 2017;31(11):3146-57. | Intervention- Not aimed to prevent weight gain during festive o holiday period |
| 94 | Pinho RAd, Petroski EL. Adiposidade corporal e nível de atividade física em adolescentes. Rev bras cineantropom desempenho hum. 1999;1(1):60-8. | Intervention- Study seems to not have an intervention. Study type- Not a RCT, nRCT or Cluster RCT |
| 95 | Pope M. Preventing Weight Gain in Children Who Are School Age and African-American. Pediatr. 2016;28(2):207-16. | Intervention - Not during festive or holiday period, just the pilot was during spring and summer. |
| 96 | Praet SF, van Rooij ES, Wijtvliet A, Boonman-de Winter LJ, Enneking T, Kuipers H, et al. Brisk walking compared with an individualised medical fitness programme for patients with type 2 diabetes: a randomised controlled trial. Diabetologia. 2008;51(5):736-46. | Participants- Study included participants with T2D |
| 97 | Pressler A, Knebel U, Esch S, Kolbl D, Esefeld K, Scherr J, et al. An internet-delivered exercise intervention for workplace health promotion in overweight sedentary employees: a randomized trial. Prev Med. 2010;51(3-4):234-9. | Intervention- Not aimed to prevent weight gain during festive or holiday period |
| 98 | Ramadan J, Telahoun G, Al-Zaid NS, Barac-Nieto M. Responses to exercise, fluid, and energy balances during Ramadan in sedentary and active males. Nutrition. 1999;15(10):735-9. | Intervention- Not aimed to prevent weight gain during festive or holiday period |
| 99 | Raman A, Ritchie LD, Lustig RH, Fitch MD, Hudes ML, Fleming SE. Insulin resistance is improved in overweight African American boys but not in girls following a one-year multidisciplinary community intervention program. J Pediatr Endocrinol Metab. 2010;23(1-2):109-20. | Intervention- Not aimed to prevent weight gain during festive or holiday period |
| 100 | Ramirez-Jimenez M, Morales-Palomo F, Ortega JF, Moreno-Cabanas A, Guio de Prada V, Alvarez-Jimenez L, et al. Effects of Exercise Training during Christmas on Body Weight and Cardiometabolic Health in Overweight Individuals. Int J Environ Res Public Health. 2020;17(13):01. | Participants- It is not clear the use of drugs for high blood preasure, since some drugs like propanolol has effect on body weight. Same in fasting blood glucose (participants with diabetes) |
| 101 | Ramirez-Rivera DL, Martinez-Contreras T, Villegas-Valle RC, Henry-Mejia G, Quizan-Plata T, Haby MM, et al. Preliminary Results of the Planet Nutrition Program on Obesity Parameters in Mexican Schoolchildren: Pilot Single-School Randomized Controlled Trial. Int J Environ Res Public Health. 2021;18(2):18. | Intervention- Not aimed to prevent weight gain during festive or holiday period |
| 102 | Ranjbar R, Ahmadizad S, Khoshniyat-Niko M, Mohsenzade A. The effect of endurance training accompanied by fasting and a period of detraining on serum leptin and fructosamine in overweight men. [Persian]. Iranian Journal of Endocrinology and Metabolism. 2013;15(3). | Intervention- Not aimed to prevent weight gain during festive or holiday period |
| 103 | Reesor L, Moreno JP, Johnston CA, Hernandez DC. School-Based Weight Management Program Curbs Summer Weight Gain Among Low-Income Hispanic Middle School Students. J Sch Health. 2019;89(1):59-67. | Study type- It is not a RCT, it is some kind of meta-analysis |
| 104 | Sarri K, Linardakis M, Codrington C, Kafatos A. Does the periodic vegetarianism of Greek Orthodox Christians benefit blood pressure? Prev Med. 2007;44(4):341-8. | Study type- Not a RCT, nRCT or a Cluster RCT |
| 105 | Sarri KO, Tzanakis NE, Linardakis MK, Mamalakis GD, Kafatos AG. Effects of Greek orthodox christian church fasting on serum lipids and obesity. BMC Public Health. 2003;3:1-8. | Study type- Not a RCT, nRCT or a Cluster RCT |
| 106 | Schneider RJ, Kusch J, Dressel A, Bernstein R. Can a twelve-week intervention reduce barriers to bicycling among overweight adults in low-income Latino and Black communities? Transportation Research Part F: Traffic Psychology and Behaviour. 2018;56:99-112. | Intervention- Not aimed to prevent weight gain during festive or holiday period |
| 107 | Shaltout I, Zakaria A, Abdelwahab AM, Hamed AK, Elsaid NH, Attia MA. Culturally based pre-Ramadan education increased benefits and reduced hazards of Ramadan fasting for type 2 diabetic patients. Journal of Diabetes and Metabolic Disorders. 2020;19(1):179-86. | Participants- Study included participants with T2D |
| 108 | Silva CS, Teixeira VH, Carvalho P. The Effect of a Nutrition Education Intervention on School-age Boys Attending a Sports Camp. Nutrícias. 2013(19):16-20. | Intervention- Duration was less than 2 weeks Other- Couldn't decide, Conference abstract |
| 109 | Silva EF, Oliveira MAd, Mendes EL, Ferreira AP, André R, Brito CJ, et al. Influência do período de férias na aptidão física de escolares. J Health Sci Inst. 2010;28(2). | Study type- Not a RCT, nRCT or a Cluster RCT |
| 110 | Silverstein LJ, Jeor STS, Harrington ME, Hayes BI, Simpson MT. Prevention of Weight Gain through the Holidays. J Am Diet Assoc. 1996;96(9, Supplement):A30. | Study type- Not a RCT, nRCT or Clúster RCT |
| 111 | Solis L, Ponte L, Martinez D, Park D, Park KS. Effects of telephone intervention on inflammatory cytokines following summer lifestyle intervention program. FASEB Journal Conference: Experimental Biology. 2015;29(Meeting Abstracts). | Intervention- Not aimed to prevent weight gain during festive or holiday period |
| 112 | Stookey JD, Evans J, Chan C, Tao-Lew L, Arana T, Arthur S. Healthy apple program to support child care centers to alter nutrition and physical activity practices and improve child weight: a cluster randomized trial. BMC Public Health. 2017;17(1):965. | Participants- Participants aged below 6 years |
| 113 | Tayebi SM, Niaki AG, Hanachi P, Ghaziani FGA. The effect of Ramadan fasting and weight-lifting training on plasma volume, glucose and lipids profile of male weight-lifters. Iranian Journal of Basic Medical Sciences. 2010;13(2 SPRING):57-62. | Intervention- Not aimed to prevent weight gain during festive or holiday period |
| 114 | Tctr. Intensive lifestyle modification program on weight loss and metabolic syndrome risks among obese women in rural areas: a randomized control trial. http://wwwwhoint/trialsearch/Trial2aspx?TrialID=TCTR20160419002. 2016. | Intervention- Aimed to weight loss |
| 115 | Teixeira CGdO, Teixeira Júnior J, Venâncio PSM, França N. Nível de atividade física nos períodos de aula e de férias, em escolares de Anápolis-GO. Rev bras ciênc mov. 2005;13(1):45-9. | Study type- Not a RCT, nRCT or a Cluster RCT |
| 116 | Trabelsi K, el Abed K, Stannard SR, Jammoussi K, Zeghal KM, Hakim A. Effects of fed- versus fasted-state aerobic training during Ramadan on body composition and some metabolic parameters in physically active men. Int J Sport Nutr Exerc Metab. 2012;22(1):11-8. | Intervention- Not aimed to prevent weight gain during festive or holiday period |
| 117 | Unalacak M, Kara IH, Baltaci D, Erdem O, Bucaktepe PG. Effects of Ramadan fasting on biochemical and hematological parameters and cytokines in healthy and obese individuals. Metab. 2011;9(2):157-61. | Study type- Not a RCT, nRCT or a Cluster RCT |
| 118 | Weaver RG, Armstrong B, Adams E, Beets M, White J, Flory K, et al. Feasibility & Preliminary Efficacy of Structured Programming and a Parent Intervention to Mitigate Accelerated Summer BMI Gain: A pilot study. Research square. 2022. | Study type - Not an RCT, non-RCT or Cluster design. |
| 119 | Weaver RG, Armstrong B, Hunt E, Beets MW, Brazendale K, Dugger R, et al. The impact of summer vacation on children's obesogenic behaviors and body mass index: a natural experiment. Int. 2020;17(1):153. | Study type - Not an RCT, non-RCT or Cluster design. |
| 120 | Weaver RG, Beets MW, Brazendale K, Brusseau TA. Summer Weight Gain and Fitness Loss: Causes and Potential Solutions. American Journal of Lifestyle Medicine. 2018;13(2):116-28. | Study type- Not a RCT, nRCT or Clúster RCT |
| 121 | Weaver RG, Brazendale K, Chandler JL, Turner-McGrievy GM, Moore JB, Huberty JL, et al. First year physical activity findings from turn up the HEAT (Healthy Eating and Activity Time) in summer day camps. PLoS ONE. 2017;12(3):e0173791. | Intervention - Not aimed to prevent weight gain during festive period |
| 122 | Weaver RG, Hunt E, Rafferty A, Beets MW, Brazendale K, Turner-McGrievy G, et al. The potential of a year-round school calendar for maintaining children's weight status and fitness: Preliminary outcomes from a natural experiment. J. 2020;9(1):18-27. | Study type- Not a RCT, nRCT or a Cluster RCT |
| 123 | Weintraub M, Ginsberg G, Stein EC, Sundaresan PR, Schuster B, O'Connor P, et al. Phenylpropanolamine OROS (Acutrim) vs. placebo in combination with caloric restriction and physician-managed behavior modification. Clin Pharmacol Ther. 1986;39(5):501-9. | Intervention- Aimed to weight loss |
| 124 | Wilson MG, Padilla HM, Meng L, Daniel CN. Impact of a workplace holiday weight gain prevention program. Nutrition and Health. 2019;25(3):173-7. | Study type- Not a RCT, nRCT or Clúster RCT |
| 125 | Wyatt K, Lloyd J, Creanor S, Green C, Dean SG, Hillsdon M, et al. Cluster randomised controlled trial and economic and process evaluation to determine the effectiveness and cost effectiveness of a novel intervention [Healthy Lifestyles Programme (HeLP)] to prevent obesity in school children. Public Health Research. 2018;1:1. | Intervention- Not aimed to prevent weight gain during festive or holiday period |
| 126 | Yin Z, Moore JB, Johnson MH, Vernon MM, Gutin B. The impact of a 3-year after-school obesity prevention program in elementary school children. Child. 2012;8(1):60-70. | Intervention- Not aimed to prevent weight gain during festive or holiday period |
| 127 | Yoshinaga M, Ogata H, Aoki M, Ito Y, Hamajima T, Miyazaki A, et al. Efficacy of walking as a lifestyle modification approach for childhood obesity. A randomized controlled trial. European Heart Journal. 2016;37 (Supplement 1):248. | Intervention- Aimed to weight loss |
| 128 | Zouhal H, Bagheri R, Ashtary-Larky D, Wong A, Triki R, Hackney AC, et al. Effects of Ramadan intermittent fasting on inflammatory and biochemical biomarkers in males with obesity. Physiol Behav. 2020;225:113090. | Intervention- Not aimed to prevent weight gain during festive or holiday period |
| 129 | Zouhal H, Bagheri R, Triki R, Saeidi A, Wong A, Hackney AC, et al. Effects of Ramadan Intermittent Fasting on Gut Hormones and Body Composition in Males with Obesity. Int J Environ Res Public Health. 2020;17(15):03. | Intervention- Not aimed to prevent weight gain during festive or holiday period |

# Table S2. Intervention details for included studies.

| **Study ID; Country, references^a^** | **Intervention – description, interventionist, control, setting, use of theory** |
| --- | --- |
| **Children, Holiday - summer break** | |
| Baranowski 2003; USA^28^ | **Intervention group:** Girls health Enrichment Multi-site Studies (GEMS) Fun, Food, and Fitness Project (FFFP): special 4-week summer day camp, followed by a special 8-week home Internet intervention for the girls and their parents - both with GEMS-FFFP enhancements.  **Interventionist:** Not reported. **Control group:** 4-week summer day camp, followed by a monthly home Internet intervention without GEMS-FFFP enhancements. **Setting:** Day camp. **Use of theory:** Social cognitive theory. |
| Evans, 2018; USA^29,40^ | **Intervention group:** Four hours a day, from Monday through Friday, for 8-weeks over the 2016 summer. Intervention components included a minimum of two hours of physical activity programming (Sports, Play Active Recreation for Kids After School - SPARK AS) and lunch offered through the U.S. Department of Agriculture’s Summer Food Service Program (SFSP). **Interventionist:** College-age summer staff. **Comparison group:** Had SFSP access at their housing community but no access to the intervention programming. **Setting:** Community public park. **Use of theory:** Social cognitive theory. |
| Evans, 2020; USA^30,41^ | **Intervention group:** Children received 7- (2017) or 8-weeks (2018) of 7.5 hours daily, day camp offered by local Boys and Girls Clubs (BGC) in each community. The BGC provided daily transportation from each housing community to the camp. Children were grouped by age (6–8 years, and 9–12 years) and assigned to a counselor. Each day counselors led campers through physical activities, including sports, arts and crafts in 45–60 min blocks. Free breakfast and lunch meals were provided to campers daily via the U.S. Department of Agriculture’s SFSP.  **Interventionist:** None reported. **Comparison group:** Were asked to experience summer vacation as otherwise planned by their parent / guardian with nor planned to enroll in a summer day camp or other daily structured summer programming for more than 1 week over the summer. **Setting:** Day camp.  **Use of theory:** None reported. |
| Hopkins, 2019; USA^33,46,47,58^ | **Standard Care:** Nutrition and physical activity programming (CATCH nutrition education and CATCH physical education) with access to free meals and safe play. **Enhanced Care: N**utrition, physical activity, and mental health programming (CATCH nutrition education, CATCH physical education, COPE mental health curriculum, youth-mentor assisted education and caregiver engagement) with access to free meals and safe play.  **Interventionist:** None reported.  **Active Control:** Access to free meals and safe play (no nutrition, physical activity or mental health programming). **Setting:** Public schools. **Use of theory:** Social cognitive theory. |
| Kilanowski, 2015; USA^35^ | **Intervention group:** Nutrition and physical activity through calisthenics and sport lessons to migrant children, provided by a part-time media teacher in one classroom devoted to the intervention. **Interventionist:** Part-time media teacher, pediatric nurse practitioner. **Control group:** The comparison group received no other instruction with the exception of bilingual healthy eating, low-literacy, publicly available CDC flyers on healthy eating that were distributed to students in class and sent home in students’ backpacks. **Setting:**  Midwest Migrant Education Program locations.  **Use of theory:** None reported. |
| Meucci, 2013; USA^36^ | **Intervention group:** The 4-week group participated in the first 4 weeks of the total eight week program. The 8-week group continued the play-based activity for 8 weeks. This program aimed to teach children new skills, to let them experiment with a wide variety of activities, and to increase strength, flexibility and cardiovascular fitness through moderate intensity activities. Hypo-caloric diets or changes in diet habits were not provided, only nutrition classes and healthy snacks and lunches were given during the program.  **Interventionist:** Expert instructors. **Control group:** Followed their usual summer break without any intervention from the study coordinators; however, they were asked to maintain their current level of physical activity for the duration of the study. **Setting:** Day camp. **Use of theory:** Social cognitive theory. |
| von Klinggraeff, 2022;  USA^38,39,48^ | **Healthy Summer Learners (HSL)**: 6-week program, 4 days per week of 7.5 hours, designed to (1) address summer declines in reading achievement and (2) mitigate accelerated unhealthy BMI gains. Alternated academic classes with physical activity, with 15-minute nutrition education session during lunch, plus healthy breakfast, lunch and snack. **21st Century Summer Learning Program (21C)**: 4- or 6-week program, 4 days per week of 5.5 hours, providing academic enrichment opportunities. Included 3.25 hours of academic sessions plus 1 hour of physical activity before lunch. Healthy breakfast and lunch.  **Interventionist:** None reported. **Control:** No program.  **Setting:** School. **Use of theory:** Social cognitive theory. |
| **Adults - Winter holiday (or Chilean National Holidays - Hernandez-Jaña 2020)** | |
| Hernandez-Jaña, 2020; Chile^31^ | **Intervention group:** Received an intervention emulating a traditional nutritional session from a primary healthcare center (public health system). One session that lasted around 20 minutes and included a body composition measurement, nutritional assessment, and a brief educational talk about healthy eating. This group received a series of healthy recommendations specially focused on the Chilean National Holidays.  **Interventionist:** None reported. **Control group:** Were asked to continue their normal activities. **Setting:** University laboratory.  **Use of theory:** None reported. |
| Hirsh, 2019; USA^32,42^ | **Intervention group:** Nutrition program consisted of 52 days of intermittent energy restriction. On two consecutive days (Monday and Tuesday) of each week, participants decreased their energy intake (730 kcal/day; 3050 kJ/day) by consuming a commercially available shake (170 kcal) four times per day. On the remaining 5 days (Wednesday to Sunday) participants were instructed to eat their habitual diet with no specific dietary recommendations. The nutrition program also included the daily consumption of a set of commercially available dietary supplements.  **Interventionist:** None reported. **Control group:** Were required to follow their habitual diet without any restriction and take one tablet of a commercially available multivitamin daily for 52 days.  **Setting:** Clinical setting.  **Use of theory:** None reported. |
| Kaviani, 2019; USA^34,43^ | **Intervention group:** Were told to perform daily self-weighing (DSW). They were asked to start DSW at the beginning of Thanksgiving week of 2017 until the week of 2018's New Year’s Day. They weighed themselves first thing in the morning after voiding (and after defecating if that was their normal pattern). The average of the first 4 days of body weight served as the “baseline” weight, which was then set as the participant’s “target” weight. Participants were instructed to try not to gain weight above this target line, and no additional instructions on how to achieve that goal were provided.  **Interventionist:** None reported. **Control group:** Participants did not receive any intervention; however, they completed the same study visits as the intervention group. **Setting:** Clinical setting. **Use of theory:** Social cognitive theory. |
| Mason, 2018; UK^16,44^ | **Intervention group:** Aimed to increase restraint of eating and drinking through regular self-weighing and recording of weight and reflection on weight trajectory; providing information on good weight management strategies over the Christmas period; and pictorial information on the physical activity calorie equivalent (PACE) of regularly consumed festive foods and drinks. The goal was to gain no more than 0.5 kg of baseline weight.  **Interventionist:** None reported. **Comparison group:** Received a leaflet on healthy living. **Setting:** home or convenient location. **Use of theory:** Self-regulation theory and habit formation model. |
| Watras, 2007; USA^37^ | **Intervention group:** 4 g/day of 78% active CLA isomers of safflower oil (3.2 g/day CLA).  **Interventionist:** None reported. **Comparison group:** 4 g/day of placebo (safflower oil). Each supplement was prepared from the same lot and capsules were identical in color, size and taste. Subjects were instructed to take four soft gel capsules each morning with food. **Setting:** Clinical setting.  **Use of theory:** None reported. |
| **Abbreviations:** BMI: Body Mass Index, BMIz: BMI z-score, BMIp: BMI percentiles, CAMP: Camp Day, CATCH: Coordinated Approach to Child Health, CLA: Conjugated Linoleic Acid, COPE: Creating Opportunities for Personal Empowerment, FM: Fat Mass, SAU: Summer as Usual, RCT: Randomized Controlled Trial, UK: United Kingdom, USA: United States of America.  **^a^** For studies with more than one reference listed, the first is the primary reference. | |

# Figure S1. Risk of bias assessment in included studies.

**
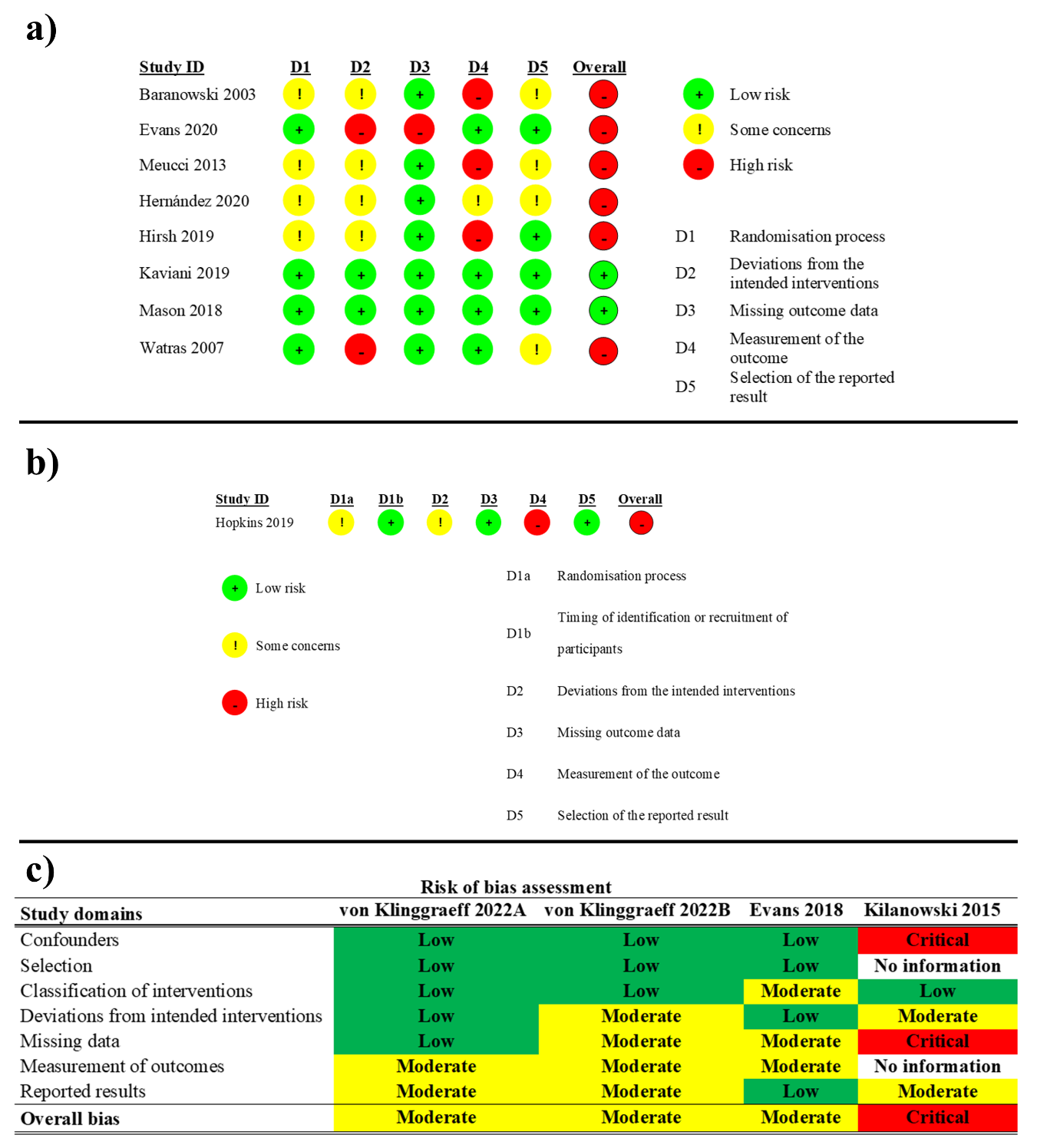
**

# Figure S1a. Individual risk of bias assessment in randomized controlled trials. *Note:* the top three studies were in children and adolescents (Baranowski 2003, Evans 2020, Meucci 2013) and the bottom five studies were in adults. S1b. Individual risk of bias assessment in cluster randomized controlled trial. *Note:* this study was conducted with children. S1c. Individual risk of bias assessment in non-randomized controlled trials.

# Table S3. Key findings of included studies.

| **Study ID; country and references^a^** | **Risk of bias^b^** | **Key findings** |
| --- | --- | --- |
| **Children, Holiday - summer break** | | |
| Baranowski 2003; USA^31^ | High risk | After adjusting for baseline BMI as a covariate, BMI at 12 weeks did not vary between groups - adjusted difference 0.6 (SE 1.6) kg/m^2^, P=0.72 (favoring the control group). After adjusting for baseline values as a covariate, waist circumference at 12 weeks did not vary between groups - adjusted difference 2.4 (SE 1.4) cm, P=0.1. Results of fat mass at follow-up were not reported. |
| Evans, 2018; USA^32, 43^ | Moderate risk^c^ | There was a non-significant 0.07 unit difference in change in BMIz between the intervention and comparison groups (− 0.04 ± 0.21 vs. 0.03 ± 0.13 BMIz units; p = 0.07). After adjusting for clustering by family and controlling for group differences in age and race/ethnicity (β = −0.10; p = 0.07) the results showed no statistical differences between groups. |
| Evans, 2020; USA^33, 44^ | High risk | CAMP participants lost − 0.03 ± 0.03 BMIz units while those in SAU gained 0.07 ± 0.03 BMIz units over the summer (β = 0.10; p = .02). These are adjusted means from linear mixed model, adjusted for clustering by family and controlling for year, age, race/ethnicity and baseline values. |
| Hopkins, 2019; USA^36, 49, 50, 64^ | High risk^d^ | Overall mean change in BMIz units for Enhanced Care group was 0.03, SE= 0.05, for Standard Care group -0.05, SE= 0.12, and for Active Control group -0.11, SE= 0.07; change in BMIz units did not differ significantly between treatment group. |
| Kilanowski, 2015; USA^38^ | Critical risk^c^ | Statistically significant were: increase in comparison group mean weight and decrease in intervention group BMIp. The intervention students showed trends towards healthy BMI. |
| Meucci, 2013; USA^39^ | High risk | The results showed no significant changes in body weight and body composition in any of the three groups after the 4 and 8-week period (p value = 0.1, eta^2^ = 0.056). However, % body fat decreased by 7.3% and 6.7% in the 4-week and 8-week groups, respectively, whereas the control group did not change after the study period. |
| von Klinggraeff, 2022;  USA^41, 42, 51^ | Moderate risk^c^ | Over the summer intervention period, control group participants experienced a statistically significant increase in BMIz units of 0.06 (95%CI=0.01, 0.12), while HSL (Δ=0.03, 95%CI=−0.03, 0.10) and 21 C (Δ=0.02, 95%CI=−0.03, 0.08) groups did not. Significant differences between HSL and 21C vs control participants were not detected. At the 12-month follow-up the change in BMIz units was greater for control group (Δ 0.09, 95%CI=0.01, 0.12) than HSL (Δ 0.04, CI95%=-0.02, 0.14) and 21C (Δ 0.04, CI95%=-0.03, 0.10). Significant differences between HSL and 21C vs control participants were not detected. |
| **Adults - Winter holiday (or Chilean National Holidays - Hernandez-Jaña 2020)** | | |
| Hernandez-Jaña, 2020; Chile^34^ | High risk | Weight increased in the IG, but decreased in the CG (IG = 0.42 kg, SD = 1.23; CG = -0.27, SD = 0.72; ∆ =-0.697, p = 0.116 [95% CI = -1.5814, 0.1860], favoring CG). FM increased significantly in the CG, but not in the IG (CG = 428.1 g; IG = 321.9 g; ∆ = 106.2 g; p = 0.654 [95% CI = −379.57, 591.92]). However, no differences were found during the NH between them (Hedges’ g effect size = 0.19; p = 0.654). |
| Hirsh, 2019; USA^35, 45^ | High risk | After 52 d, intervention participants lost a total of 1·3 kg (1·7 %) from baseline (75·0 (SD 9·8) v. 76·3 (SD 9·8) kg; P < 0·05). Subjects in the control group lost 0·3 kg from baseline at day 24 and 0·4 kg at day 52, which was not statistically significant. There was no significant between-group difference in weight loss at day 24 or day 52. Intermittent gastrointestinal adverse effects, with mild to moderate severity, were reported by 20% of intervention group participants. |
| Kaviani, 2019; USA^37, 46^ | Low risk | Weight change during the  7-week holiday period was greater for control versus DSW + GF (2.65 ± 0.33 vs. −0.13 ± 0.27 kg, respectively; P < 0.001) and over the entire study period (1.51 ± 0.39 vs. −0.15 ± 0.35 kg for control vs. DSW + GF, respectively; P = 0.002), which was 21 weeks from baseline.  There was a treatment visit interaction for total body fat percentage (TBF%; P = 0.001) with a greater decrease in DSW + GF versus control during the holidays (−1.08% ± 0.19% vs. 0.95% ± 0.19%, respectively; P < 0.001) and throughout the entire study (−0.87% ± 0.37% vs. 0.45% ± 0.26%, respectively; P = 0.01). No significant difference between groups was found in waist circumference. |
| Mason, 2018; UK^19, 47^ | Low risk | Mean weight change was −0.13 kg (95% confidence interval −0.4 to 0.15) in the intervention group and 0.37 kg (0.12 to 0.62) in the comparator group. The adjusted mean difference in weight (intervention− comparator) was −0.49 kg (95% confidence interval −0.85 to −0.13, P=0.008). |
| Watras, 2007; USA^40^ | High risk | Compared to CLA, the placebo group showed a greater rate of weight gain during the holiday season (1.1 kg, SD 3.2 for placebo and -0.6 kg, SD 2.5 for CLA, p value < 0.05). Six-month change in % body fat was improved with CLA compared to placebo (0.2 FM%, SD 2.3 for placebo and -1.0 FM%, SD 1.8 for CLA, p value < 0.02). The rate of reported negative emotions decreased significantly with CLA, although there was no difference in any other category of adverse event. |

**Abbreviations:** BMI: Body Mass Index, BMIz: BMI z-score, BMIp: BMI percentiles, CAMP: Camp Day, CG: Control Group, CI: Confidence Interval, CLA: Conjugated Linoleic Acid, DSW+GF: Daily Self-weighing + Graphic Feedback, FM: Fat Mass, IG: Intervention Group, NH: Chilean national holidays, RCT: Randomized Controlled Trial, SAU: Summer as Usual, SE: standard error; UK: United Kingdom, USA: United States of America.

**^a^** For studies with more than one reference listed, the first is the primary reference.

^b^ The RoB 2 tool for RCTs was used to assess risk of bias of all included studies, except where otherwise noted:

**^c^** ROBINS I tool used to assess non-RCT.

**^d^** RoB 2 tool for cluster-RCT.

# Figure S2. Sensitivity analysis of studies in adults – fixed effects model


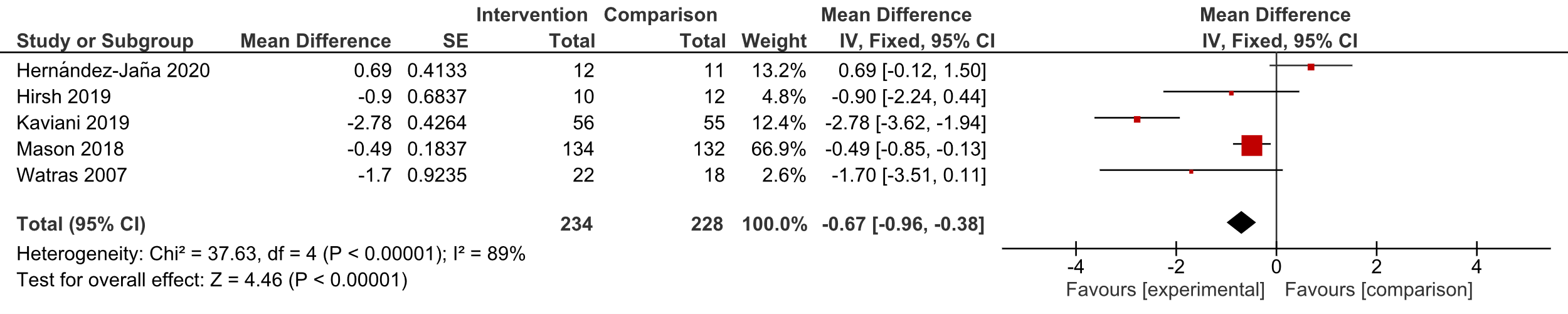


# Figure S3. Sensitivity analysis of studies in adults – low risk of bias studies


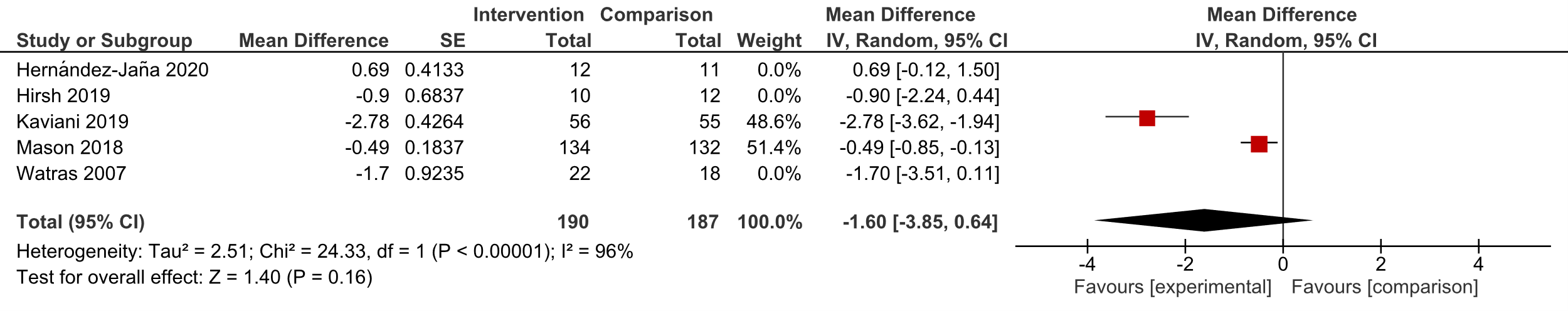


# Figure S4. Subgroup analysis for type of holiday period for studies in adults –December holiday period vs Chilean national holidays.


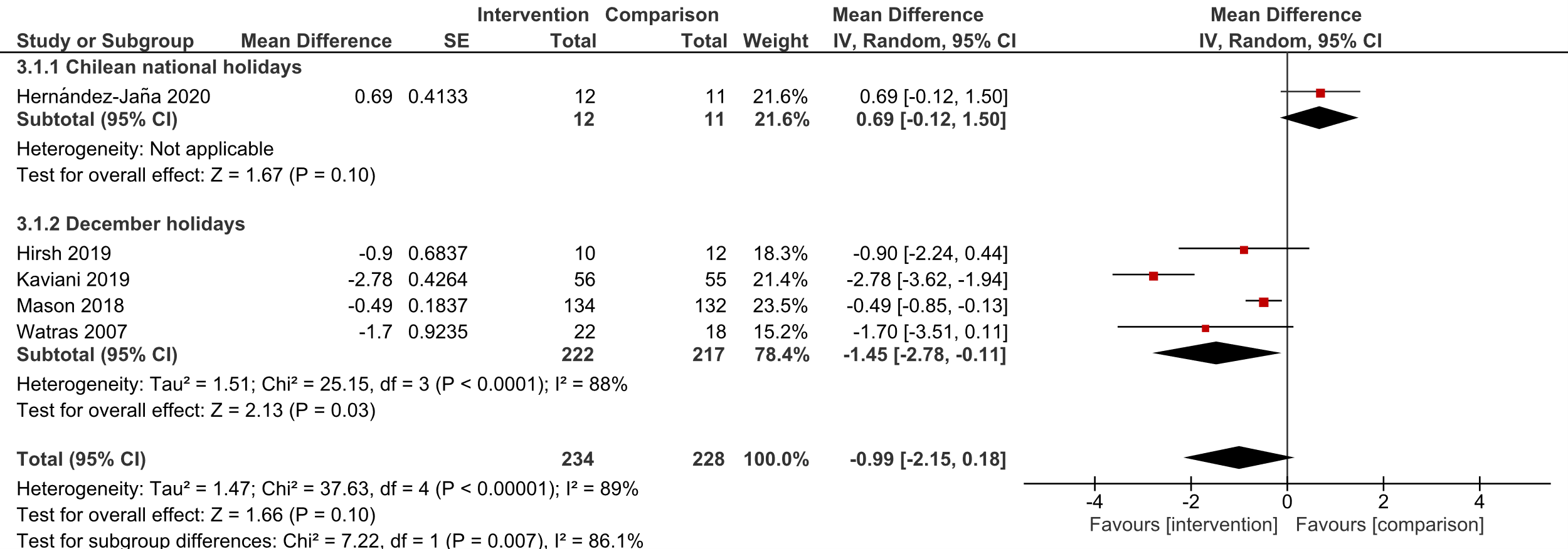


# Table S4. GRADE assessment of quality of evidence for studies in children and adolescents

| **Intervention compared to control for the prevention of weight gain during festive and/or holiday periods in children** | | | | | | | | | | | |
| --- | --- | --- | --- | --- | --- | --- | --- | --- | --- | --- | --- |
| **Certainty assessment** | | | | | | | **Summary of findings** | | | | |
| **Participants (studies) Follow-up** | **Risk of bias** | **Inconsistency** | **Indirectness** | **Imprecision** | **Other considerations** | **Overall certainty of evidence** | **Study event rates (%)** | | **Relative effect (95% CI)** | **Anticipated absolute effects** | |
|  |  |  |  |  |  |  | **With [Comparison]** | **With [Intervention]** |  | **Risk with [Comparison]** | **Risk difference with [Intervention]** |
| **BMI-Z-Score** | | | | | | | | | | | |
| 423 (4 observational studies) | very serious^a^ | serious^b^ | not serious | not serious^c^ | none | ⨁◯◯◯ Very low | 153 | 270 | - | The mean BMI z-score was **0** | MD **0.06 lower** (0.1 lower to 0.01 lower) |

**CI:** confidence interval; **MD:** mean difference

*Explanations*

a. The study designs included 1 randomized controlled trial, 1 cluster-randomized controlled trial and two non-randomized controlled trials. Of the four included studies, two were classified as moderate risk of bias and two as high risk of bias.

b. Although the heterogeneity test showed no heterogeneity (I^2^= 0%), we observed variability of effect estimates, with one study (two intervention arms) showing a negative effect of the intervention.

c. Optimal information size (OIS) criterion was met with a calculated sample size of 338 participants using data from the study by Evans 2020. Additionally, the 95% CI excludes a mean difference of 0.

# Table S5. GRADE assessment of quality of evidence for studies in adults

| **Intervention compared to control for the prevention of weight gain during festive and/or holiday periods in adults** | | | | | | | | | | | |
| --- | --- | --- | --- | --- | --- | --- | --- | --- | --- | --- | --- |
| **Certainty assessment** | | | | | | | **Summary of findings** | | | | |
| **Participants (studies) Follow-up** | **Risk of bias** | **Inconsistency** | **Indirectness** | **Imprecision** | **Publication bias** | **Overall certainty of evidence** | **Study event rates (%)** | | **Relative effect (95% CI)** | **Anticipated absolute effects** | |
|  |  |  |  |  |  |  | **With [Comparison]** | **With [Intervention ]** |  | **Risk with [Comparison]** | **Risk difference with [Intervention ]** |
| **Body weight follow-up** | | | | | | | | | | | |
| 462 (5 RCTs) | serious^a^ | serious^b^ | serious^c^ | serious^d^ | none | ⨁◯◯◯ Very low | 228 | 234 | - | The mean body weight follow-up was **0** | MD **0.99 lower** (2.15 lower to 0.18 higher) |

**CI:** confidence interval; **MD:** mean difference

*Explanations*

a. Of the five studies evaluated with the RoB2 tool, three were classified with high risk of bias and two were classified with low risk of bias.

b. A meta-analysis showed inconsistency in results due to substantial heterogeneity (I^2^=89%). Although not previously specified, a sub-group analysis for type of holiday period (December holiday period vs Chilean national holidays) showed a significant overall effect of the intervention in the December holiday period: -1.45 kg (95% CI [-2.78, -0.11], P= 0.03; I^2^= 88%) with moderate heterogeneity.

c. There are some differences in population and interventions between studies. Three studies involved USA population, one from UK and another one from Chile. Interventions were in different settings (e.g. Northern vs Southern hemisphere). However, all studies were implemented during the winter season. We did not have any concern about differences in outcome since every study reported changes in body weight and we did not define an end point of interest.

d. Optimal information size (OIS) criterion was met, since we estimated a sample size of 160 using standard deviation from one of the relevant studies (Mason) and obtained a sample size of 432 for this outcome analysis. However, 95% confidence interval overlaps no effect (i.e. CI includes mean difference 0).
